# A bioinformatics screen reveals hox and chromatin remodeling factors at the *Drosophila* histone locus

**DOI:** 10.1186/s12863-023-01147-0

**Published:** 2023-09-21

**Authors:** Lauren J. Hodkinson, Connor Smith, H. Skye Comstra, Bukola A. Ajani, Eric H. Albanese, Kawsar Arsalan, Alvaro Perez Daisson, Katherine B. Forrest, Elijah H. Fox, Matthew R. Guerette, Samia Khan, Madeleine P. Koenig, Shivani Lam, Ava S. Lewandowski, Lauren J. Mahoney, Nasserallah Manai, JonCarlo Miglay, Blake A. Miller, Olivia Milloway, Nhi Ngo, Vu D. Ngo, Nicole F. Oey, Tanya A. Punjani, HaoMin SiMa, Hollis Zeng, Casey A. Schmidt, Leila E. Rieder

**Affiliations:** 1 Genetics and Molecular Biology graduate program, Emory University, Atlanta, GA 30322 USA; 2 Department of Biology, Emory University, 1510 Clifton Road, Atlanta, GA 30322 USA

**Keywords:** *Drosophila*, ChIP-seq, Galaxy, Course-based Undergraduate Research Experience, Hox factors, Histone locus, Histone locus body

## Abstract

**Background:**

Cells orchestrate histone biogenesis with strict temporal and quantitative control. To efficiently regulate histone biogenesis, the repetitive *Drosophila melanogaster* replication-dependent histone genes are arrayed and clustered at a single locus. Regulatory factors concentrate in a nuclear body known as the histone locus body (HLB), which forms around the locus. Historically, HLB factors are largely discovered by chance, and few are known to interact directly with DNA. It is therefore unclear how the histone genes are specifically targeted for unique and coordinated regulation.

**Results:**

To expand the list of known HLB factors, we performed a candidate-based screen by mapping 30 publicly available ChIP datasets of 27 unique factors to the *Drosophila* histone gene array. We identified novel transcription factor candidates, including the *Drosophila* Hox proteins Ultrabithorax (Ubx), Abdominal-A (Abd-A), and Abdominal-B (Abd-B), suggesting a new pathway for these factors in influencing body plan morphogenesis. Additionally, we identified six other factors that target the histone gene array: JIL-1, hormone-like receptor 78 (Hr78), the long isoform of female sterile homeotic (1) (fs(1)h) as well as the general transcription factors TBP associated factor 1 (TAF-1), Transcription Factor IIB (TFIIB), and Transcription Factor IIF (TFIIF).

**Conclusions:**

Our foundational screen provides several candidates for future studies into factors that may influence histone biogenesis. Further, our study emphasizes the powerful reservoir of publicly available datasets, which can be mined as a primary screening technique.

**Supplementary Information:**

The online version contains supplementary material available at 10.1186/s12863-023-01147-0.

## Introduction

Cells rely on strict temporal and quantitative orchestration of gene expression. One way the nucleus accomplishes coordinated gene regulation is through the establishment of nuclear bodies (NBs), membraneless concentrations of proteins and RNAs. The NB micro-environment facilitates processes such as efficient gene expression through transcription and RNA processing [1–3].

**Fig. 1:**
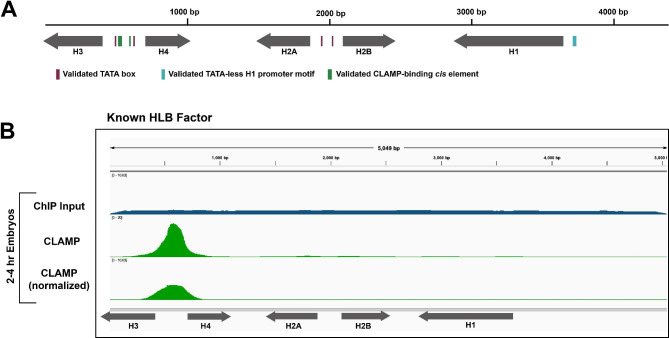
Known HLB factor CLAMP localizes to the GA-repeat *cis* elements in the *H3/H4 *promoter. **(A)** A diagram detailing the validated *cis* elements in the histone array including the TATA-box elements (maroon boxes), the TATA-less motif (teal box), and the CLAMP binding GA-repeat elements (green boxes). **(B)** We mapped ChIP-seq data for the known HLB factor CLAMP (green) from 2–4 h embryos [12]. The ChIP signal was normalized to its respective ChIP input signal (blue)

The histone locus body (HLB) is a conserved NB that regulates histone gene expression and forms at the loci of the replication-dependent histone genes [4] in many different organisms, including humans and *Drosophila*. The HLB is characterized by a set of factors that collectively regulate the uniquely organized histone genes. The *Drosophila melanogaster* histone locus is a cluster of ~ 100 tandemly repeated arrays, in which each 5 Kb array includes the 5 canonical histone genes along with their respective promoters and regulatory elements [4–6]. Each array contains two TATA-box containing promoters, one for *H3* and *H4* and one for *H2A* and *H2B* (Fig. 1A). Additionally, the *H1* gene has its own unique promoter that lacks a TATA-box. The promoters contain some known transcription factor motifs [7–9], but overall little is known about how the locus is transcriptionally controlled. The clustered, repetitive organization of the locus allows for precise HLB formation at a single genomic location and highly coordinated histone biogenesis linked to S-phase of the cell cycle [10, 11].

The *Drosophila* HLB is a well-characterized NB that includes several known components that play a role in both the cell cycle regulation of histone gene transcription and the unique processing of histone mRNA transcripts. Several proteins are involved in the initiation and regulation of histone gene transcription including Chromatin Linked Adaptor for MSL proteins (CLAMP [9, 12]); Multi Sex combs (Mxc [11, 13]), the *Drosophila* ortholog of human Nuclear Protein mapped to the Ataxia-Telangiectasia locus (NPAT [14]); FLICE-associated huge protein (FLASH [2]); and Muscle wasted (Mute [15]). Histone mRNA processing is distinct from that of other mRNAs because histone pre-mRNAs lack polyA tails and introns [4]. Several known factors are involved in histone mRNA processing and target the histone gene locus, including the U7 snRNP [16], Stem Loop Binding Protein (SLBP [17]), and Lsm11 [4].

Other than CLAMP, the above-mentioned factors target the histone locus but do not interact directly with DNA sequence. Since CLAMP is found at locations genome-wide, it is currently unclear how non-DNA binding factors identify and target the histone locus. The presence of histone mRNA is likely to play a role [18] as is the presence of *cis* elements within the histone gene array [9, 19]. One critical interaction involves CLAMP recognizing GA-repeat sequences within the *H3/H4* promoter [9] (Fig. 1**)**. Although the presence of CLAMP is critical for the localization of HLB-specific factors such as Mxc [9], the interaction between CLAMP and GA-repeats is not strictly necessary for HLB formation [20] and CLAMP is not sufficient for HLB formation [9]. Therefore, it is likely that other DNA-interacting proteins participate in defining the histone locus. We still lack a comprehensive list of factors associated with histone biogenesis and therefore our model of the mechanisms of histone gene regulation remains incomplete.

Historically, novel HLB factors are often discovered by chance through immunofluorescence, such as CLAMP [9], Myc [21], Mute [15], and Abnormal oocyte [22]. To discover novel DNA-binding proteins that target the histone locus, we first screened the literature for likely candidates and then funneled these into a secondary bioinformatics screen. We leveraged publicly available *Drosophila* ChIP-seq datasets and knowledge of histone gene regulation to curate and analyze a list of candidate DNA-binding factors. We used a bioinformatics pipeline on Galaxy [23, 24] to map candidate ChIP-seq data to a single copy of the histone gene array. The ~ 100 histone gene arrays are nearly identical in sequence [6] and we can collapse -omics data from the entire locus onto a single array [5, 9, 20]. Supervised undergraduate students conducted much of the initial screen as part of a course-based undergraduate research experience (CURE; [25]), demonstrating the simplicity and versatility of the pipeline design. Using our qualitative analysis criteria (Supplemental Fig. 1), we discovered several DNA-interacting proteins that pass our initial bioinformatics screen. Our novel candidates that target the histone gene array include developmental transcription factors such as Hox factors, which may provide a mechanistic link between segment identity and cell division.

Future wet lab studies are required to confirm the presence of these candidates at the histone locus, determine any tissue and temporal specificity, and describe the precise roles of candidates in HLB formation and histone biogenesis. As a whole, our screen establishes mining of existing -omics data as a tool to identify new candidate HLB factors. Although we are limited by the factors, tissues, treatments, and timepoints interrogated by the dataset generators, our pipeline is an inexpensive and rapid tool to screen candidate factors for future wet-lab studies.

## Methods

### GEO datasets

All datasets were downloaded from the NCBI SRA Run Selector through the Gene Expression Omnibus (GEO). See Table 1 for Accession numbers and references.

**Table 1:** DNA-binding factor candidate datasets

Candidate		GEO Accession #	SRA Run Selector #	Paper citation
Abd-A Abdominal-A		GSE69796	**anti-GFP ChIP DNA from Kc167 cells expressing AbdA-GFP** **1**- SRR2060648 **2 -**SRR2060649 **Input** **1 -** SRR2060652 **2 -** SRR2060653	[63]
Abd-B Abdominal-B		GSE69796	**anti-GFP ChIP DNA from Kc167 cells expressing AbdB-GFP** **1**- SRR2060650 **2 -**SRR2060651 **Input** **1 -** SRR2060652 **2 -** SRR2060653	[63]
ANTP Antennapedia		GSE125604	**anti-GFP (Invitrogen) from ANTP-GFP genotype** **1 -** SRR8483063 **Input** **1 -** SRR8483064	[61]
CP190 Centrosomal protein 190kD		GSE118699	**CP190 rabbit (Pai et al. 2004)** **1 -** SRR7706256 **2 -** SRR7706258 **Input** **1 -** SRR7706251 **2 -** SRR7706252	[40]
CTCF		GSE175402	**CTCF** **1** - SRR14631231 2 - SRR14631232 **Input** **1** - SRR14631233 2 - SRR14631234	[42]
Exd Extradenticle		GSE125604	**anti-V5 (Invitrogen) on exd-V5 transgene genotype** **1 -**SRR8483055 **Input** **1 -** SRR8483056	[61]
Fs(1)h Female sterile (1) homeotic		GSE42086	**Female late embryo-derived cell line, ChIP of Fs(1)h long isoform** **1-** SRR611533 **Female late embryo-derived cell line, ChIP of both isoforms of Fs(1)h** **1 -** SRR611535 **Input** **1 -** SRR611537	[60]
Gcn5		GSE83408	**Gcn5 rabbit polyclonal antibody (5 ug/IP)** **1 -** SRR3671294 **2 -** SRR3671295 **3 -** SRR3671298 **Input** **1 -** SRR3671296 **2 -** SRR3671297 **3 -** SRR3671299	[41]
Hr78 Hormone-receptor-like 78		GSE50370	**Hr78-GFP_8–16_embryonic_ChIP-seq_ChIP** **1 -** SRR1198798 **2 -** SRR1198799 **Input** **1 -** SRR1198796 **2 -** SRR1198797	[73]
Hnf4 Hepatocyte nuclear factor 4		GSE73675	**rat anti-dHNF4 3600** **1 -** SRR2548371 **2 -** SRR2548372 **3 -** SRR2548373 **4 -** SRR2548374 **Inputs** **1 -** SRR2548367 **2 -** SRR2548368 **3 -** SRR2548369 **4 -** SRR2548370	[53]
HTH Homothorax		GSE125604	**anti-Hth (gp52, N-terminal)** **1 -** SRR8483065 **Input** **1 -** SRR8483066	[61]
JIL-1		GSE54438	**JIL-1 monoclonal antibody 5C9** **1 -** SRR1145605 **2 -** SRR1145606 **Input** **1 -** SRR1145612 **2 -** SRR1145613	[57]
M1BP Motif 1 Binding Protein		GSE97841	**M1BP_Antibody** **1 -** SRR10759878 **Input** **1 -** SRR10759877	[30]
MSL-1 Male-specific Lethal 1		GSE37864	**polyclonal rabbit MSL1, crude serum** **1 -** SRR495366 **2 -** SRR495367 **Input** **1 -** SRR495378 **2 -** SRR495380	[39]
Ndf/CG4747 Nucleosome-destabilizing factor		GSE42025	**PAP antibody (Sigma P1291)** **1 -** SRR611192 **2 -** SRR611194 **3 -** SRR611196 **4 -** SRR611198 **Input** **1 -** SRR611193 **2 -** SRR611195 **3 -** SRR611197 **4 -** SRR611199	[29]
Nej (S2 cells) Nejire		GSE72666	**anti-CBP, custom-made antibodies** **1**- SRR2232434 **Input** **1 -** SRR2232432	[74]
Nej (Embryos) Nejire		GSE68983	**Nej** **1 -** SRR4044401 **Input** **1 -** SRR2031906	[75]
Opa Odd Paired		GSE140722	**In-house anti-Opa antibody** **1 -** SRR10502454 **2 -** SRR10502455 **3 -** SRR10502458 **4 -** SRR10502459 **Input** **1 -** SRR10502456 **2 -** SRR10502457 **3 -** SRR10502460 **4 -** SRR10502461	[51]
Pan Pangolin		GSE50340	**Pan** **1 -** SRR1198824 **2 -** SRR1198825 **Input** **1 -** SRR1198822 **2 -** SRR1198823	[73]
Pnt Pointed		GSE114092	**Pnt** **1 -** SRR7126165 **Input** **1 -** SRR7126164	[55]
Psc Posterior sex combs		GSE38166	**Psc Mitotic S2** **1** - SRR 500,149 **2** - SRR 500,150 **Psc Control S2** **1** - SRR500151 **2** - SRR500152 **Psc Mitotic S2 Input** **1** - SRR 500,153 **2** - SRR 500,154 **Psc Control S2 Input** **1** - SRR 500,155 **2** - SRR 500,156	[43]
Scm (S2 cells) Sex comb on midleg		GSE66183	**BioTAP-N-Scm** **1 -** SRR1813243 **2 -** SRR1813245 **Input** **1 -** SRR1813244 **2 -** SRR1813246	[76]
Scm (embryos) Sex comb on midleg		GSE66183	**BioTAP-N-Scm** **1 –** SRR1813233 **Input** **1 -** SRR1813234	[76]
su(z)12 suppressor of zeste 12		GSE36039	**Su(z)12 ChIP** **1 -** SRR363407 **2 -** SRR363408 **Input** **1 -** SRR363409 **2 -** SRR363410	[44]
TAF1 TBP-Associated Factor 1		GSE97841	**TAF1 Antibody** 1 - SRR5452843 2 - SRR5452844 **Inputs** 1 - SRR5452847 2 - SRR5452848	[30]
TFIIB Transcription Factor II B		GSE120152	**anti-TFIIB rabbit polyclonal, custom** **1 -** SRR7874066 **2 -** SRR7874067 **Inputs** **1 -** SRR7874069 **2 -** SR7874070	[31]
TFIIF Transcription Factor II F		GSE120152	**anti-TFIIF rabbit polyclonal, custom** **1 -** SRR7874068 **Inputs** **1 -** SRR7874069	[31]
TRF2 TBP protein-related factor 2		GSE97841	**TRF2 Antibody** **1 -** SRR5452845 **2 -** SRR5452846 **Inputs** **1 -** SRR5452847 **2 -** SRR5452848	[30]
Ubx (Kc cells) Ultrabithorax		GSE69796	**anti-GFP ChIP DNA from Kc167 cells expressing Ubx-GFP** 1 - SRR2060646 2 - SRR2060647 **Inputs**: **1 -** SRR2060652 **2 -** SRR2060653	[63]
Ubx (embryos) Ultrabithorax		GSE64284	**Anti-V5 ChIP, Ubx-V5** **1 -** SRR1721317 **2 -** SRR1721321 **Inputs** **1 -** SRR1721316 **2 -** SRR1721320	[77]
Ubx (larva) Ultrabithorax		GSE184454	**Anti-FLAG monoconal, 3xFLAG-Ubx** **1 -** SRR15972582 **2 -** SRR15972584 **Inputs** **1-** SRR15972583 **2 -** SRR15972585	[78]

### Bioinformatic analysis and data visualization

We directly imported individual FASTQ datasets into the web-based platform Galaxy [23, 24] through the NCBI SRA Run Selector by selecting the desired runs and utilizing the computing Galaxy download feature. We retrieved the FASTQ files from SRA using the “Faster Download and Extract Reads in FASTQ format from NCBI SRA” Galaxy command. Because the ~ 100 histone gene arrays are extremely similar in sequence [6], we do not utilize the dm6 or dm3 genomes and instead can collapse ChIP-seq data onto a single histone array [5, 6, 20]. We used a custom “genome” that includes a single *Drosophila melanogaster* histone array similar to that in Mckay et al. 2015, which we directly uploaded to Galaxy using the “upload data” feature, and normalized using the Galaxy command “NormalizeFasta” specifying an 80 bp line length for the output .fasta file. We aligned ChIP reads to the normalized histone gene array using Bowtie2 [26] to create .bam files using the user built-in index and “very sensitive end-to-end” parameter settings. We converted the .bam files to .bigwig files using the “bamCoverage” Galaxy command in which we set the bin size to 1 bp and set the effective genome size to user specified: 5000 bp (approximate size of l histone array). We also mapped relevant input or IgG datasets. If an input dataset was available, we normalized ChIP datasets to input using the “bamCompare” Galaxy command in which we set the bin size to 1 bp. We visualized the .bigwig files using the Integrative Genome Viewer (IGV) [27].

### Criteria for positive vs. negative candidates

Because we focused our analysis on a single 5 Kb sequence and condensed data from ~ 100 identical histone arrays onto a single array, we were unable to use quantitative peak calling programs. We instead utilized the following qualitative criteria to determine positive and negative candidates (Supplemental Fig. 1). We only considered the candidate as positive if a peak emerged in the ChIP data that was not present in the input. We considered the following false positives: (1) obvious overrepresentation of gene bodies (e.g. Su(z)12, Supplemental Fig. 2), (2) underrepresentation of intergenic regions (e.g. CP190 input, Fig. 3C), and (3) if the input coverage and ChIP coverage peaks looked identical (e.g. MSL1, Fig. 3B). Datasets with the above-mentioned characteristics cause peaks to emerge in the normalized data that do not represent the binding of the factor but rather a bias in the amplification of the ChIP library or alignment. We also checked spot length (read length) and considered peaks over the GA-repeat *cis* elements in the *H3/H4* promoter (Fig. 1A) found in datasets with read lengths $\le$50 bp false positive peaks (e.g. Psc, Supplemental Fig. 2).

**Fig. 2:**
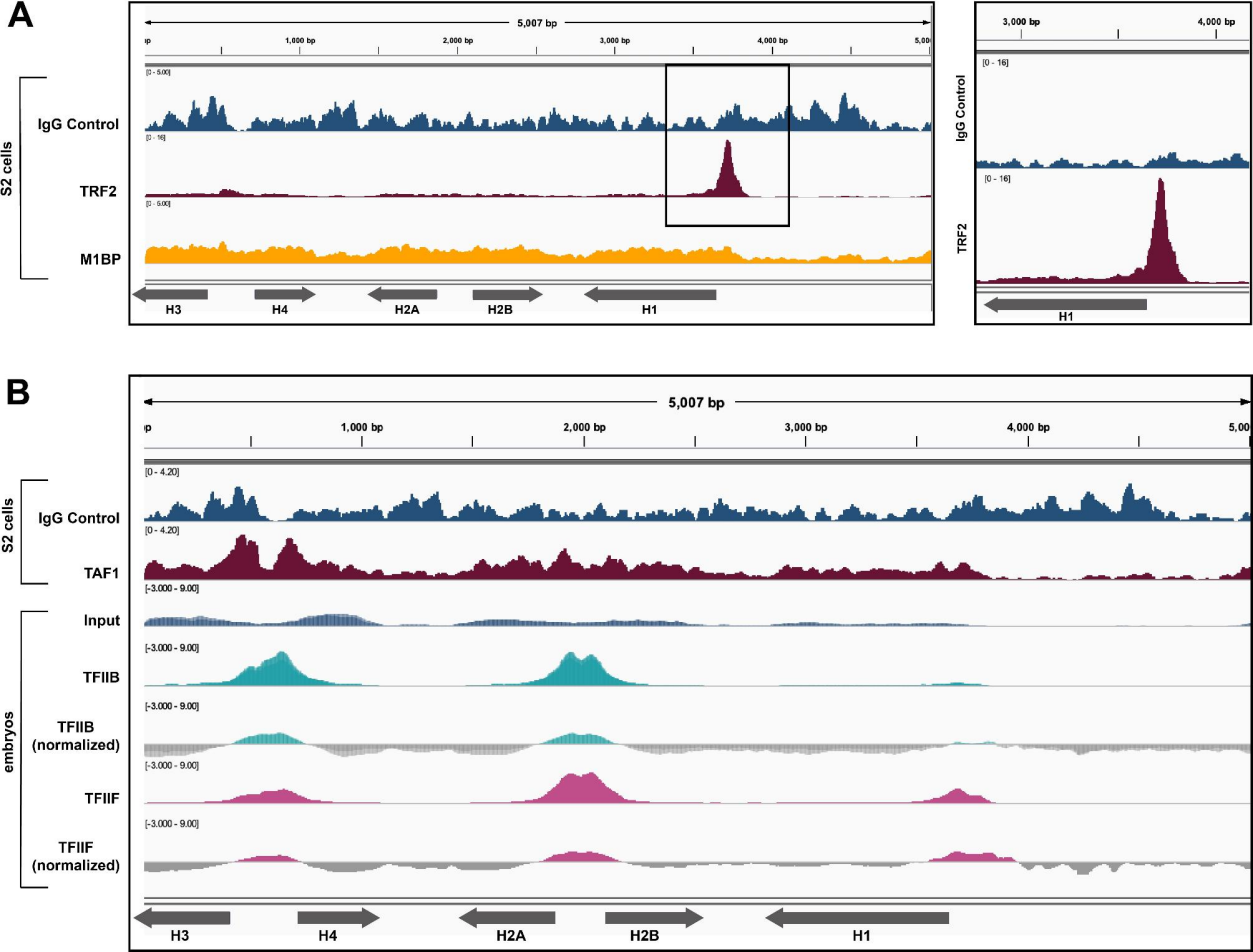
Expected general transcription factors localize to the histone array. **(A)** We mapped ChIP-exo data for TRF2 (maroon, [30]) from S2 cells to the histone gene array, which recapitulates results from Isogai et al. 2007 showing localization specifically to the *H1* promoter, validating our bioinformatics pipeline. We also mapped ChIP-exo data for M1BP (yellow, [30]) which did not localize to the histone gene array, further validating our pipeline. We compared ChIP-exo data to an IgG control (blue, [30]. **(B)** We aligned ChIP-exo data for TAF-1 (maroon, [30]) from S2 cells to the histone gene array and compared to a corresponding IgG control. We aligned ChIP-seq datasets for TFIIB (teal, two replicates overlayed, [31]) and TFIIF (pink, one replicate, [31]) from OregonR mixed population embryos to the histone gene array and normalized to the provided input (blue). TFIIB shows localization to the *H3/H4* promoter and the *H2A/H2B* promoter, and TFIIF shows localization to both core promoters and the *H1* promoter, confirming that our bioinformatics pipeline can be used to identify novel factors that localize to the histone gene array

## Results

### Validating the bioinformatics pipeline by mapping TATA-associated factors to the histone gene array

We first sought to validate our bioinformatics pipeline through analysis of known histone locus proteins and associated factors. Isogai et al. (2007) used immunofluorescence and cell culture ChIP-qPCR assays to demonstrate that the TATA binding protein (TBP)/TFIID complex selectively binds to the *H3/H4* promoter and the *H2A/H2B* promoter, but TBP-related factor 2 (TRF2) targets the promoter of the TATA-less *H1* promoter. We identified a publicly available TRF2 ChIP-exo dataset from Baumann et al. (2017) for TRF2 and used our pipeline to map the data to the histone gene array. ChIP-exo is similar to ChIP-seq but identifies a more complete set of binding locations for a factor with higher resolution than standard ChIP-seq [28]. We validated that TRF2 localizes to the *H1* promoter (Fig. 2A). Because we were unable to normalize to an input dataset, we compared the TRF2 alignment to an IgG control. The localization of TRF2 to the TATA-less *H1* promoter is consistent with Isogai et al. (2007) and is consistent with where a TBP-related factor (TRF) would be expected to bind as they are known to target TATA-less promoters [29]. Baumann et al. (2017) demonstrated that Motif 1 binding protein (M1BP) interacts with TRF2, but that this interaction is mostly restricted to the ribosomal protein genes [30]. We mapped ChIP-exo data for M1BP and observed that it did not localize to the *H1* promoter under our qualitative criteria (Supplemental Fig. 1) as we saw with TRF2, nor to any other part of the histone array (Fig. 2A), further validating our pipeline.

**Fig. 3:**
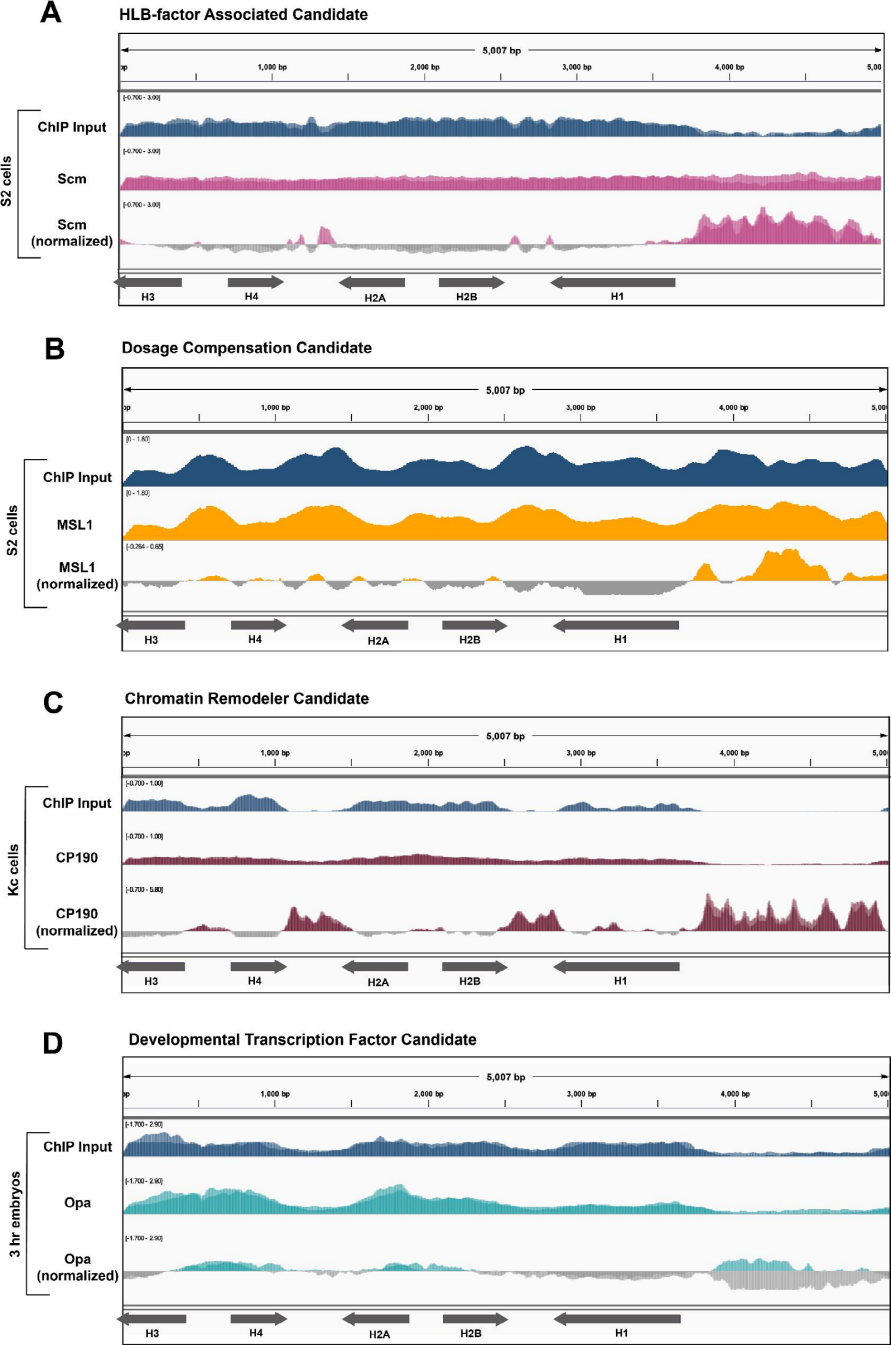
DNA-binding factors from different categories that did not pass the bioinformatics screen. We aligned ChIP-seq datasets for **(A)** Scm (pink, two replicates overlayed, [76]) from S2 cells, **(B)** MSL1 (yellow, one replicate, [39]) from S2 cells, **(C)** CP190 (maroon, two replicates overlayed, [40]) from Kc cells, and **(D)** Opa (teal, two replicates overlayed, [51]) from 3 h mixed population embryos to the histone array. We normalized each ChIP signal to its respective ChIP input signal (blue)

### Novel general transcription factors that target the histone locus

To expand the list of general transcription factors that target the histone locus, we mapped an additional ChIP-exo dataset from Baumann et al. (2017) for TAF1 (TBP associated factor 1). TAF1 is a member of the Transcription Factor IID (TFIID) complex which Isogai et al. (2007) also suggested localized to the same regions of the histone gene array as TBP. When we mapped the TAF1 ChIP-exo data, we observed that TAF1 targets the TATA-box regions of the *H3*/*H4* promoter and, less robustly, the TATA-box regions of the *H2A*/*H2B* promoter (Fig. 2B, elements annotated in Fig. 1A). Again, we compared this alignment to an IgG control because we were unable to normalize to an input, but because TAF1 associates with TBP which binds to AT-rich (TATA box) regions [30], the localization of TAF1 to the TATA-box regions of the core histone genes is expected.

To test the ability of our pipeline to identify novel factors that localize to the histone gene array, we investigated the relationships of additional general transcription factors to the histone array. We identified ChIP-seq datasets for both TFIIB and TFIIF. Both TFIIB and TFIIF are associated with TBP [31] and therefore we would expect them to localize to the *H3/H4* and *H2A/H2B* promoters, similar to TBP [8]. We observed both TFIIB and TFIIF localization to the *H3/H4* and *H2A/H2B* promoters while, surprisingly, TFIIF also localized to the *H1* promoter (Fig. 2B).

### Candidate DNA-binding factors that did not pass the bioinformatics screen

After verifying our bioinformatics pipeline, we curated a list of candidate DNA-binding factors (Table 1, Supplemental Table 1) that we hypothesized would target the histone gene array. To create this candidate list, we prioritized factors that meet at least one of the following criteria: (1) DNA-binding factors with a relationship to a validated HLB factor; (2) DNA-binding factors involved in dosage compensation, because CLAMP, a non-sex specific dosage compensation factor, targets the histone locus [9, 20] (Fig. 1B); (3) chromatin remodeling or histone-interacting factors, since the epigenetic landscape of the histone locus is largely undefined; (4) early developmental transcription factors, since histone gene regulation is critical during early development and synchronized cell division [32]. We also utilized the online platform STRING [33] that provides the known and inferred interactomes of a given protein to identify candidates that met the above criteria. Out of the 27 candidates, we rejected 19 as likely not targeting the histone gene array based on our qualitative analysis of the datasets we investigated (Supplemental Fig. 1).

### HLB factor-associated candidates

We investigated the DNA-binding factor Sex comb on midleg (Scm), because of its suspected interaction with the known HLB factor Multi-sex combs (Mxc; [11, 13]). Based on STRING, Scm is predicted to interact with Mxc, as determined by a genetic interference assay in which a double Mxc/Scm mutant resulted in enhanced mutant sex comb phenotypes [34, 35]. Despite possible interaction with Mxc, neither Scm ChIP-seq data from S2 cells (Fig. 3A**)** nor from 12 to 24 h embryos (Supplemental Fig. 2) gave meaningful signal over the histone gene array. This result was surprising because the human ortholog of Mxc (NPAT) associates exclusively with the histone promoters [36], and Mxc is only found at the histone locus [14].

### Dosage compensation candidates

The HLB factor CLAMP targets the *H3/H4* promoter (Fig. 1B) and regulates histone gene expression [9], but also plays additional roles in *Drosophila* male dosage compensation: it binds to GA-rich elements along the male X-chromosome and recruits the Male Specific Lethal complex (MSLc). Further, MSL2, the male specific component of MSLc, also emerged from a cell-based HLB factor screen [11], and we recently discovered that MSL2 targets one histone gene locus in *Drosophila virilis* [37]. We therefore hypothesized that dosage compensation factors target the histone gene array along with CLAMP. We chose the following DNA-binding factors for our candidate screen because of their relationship to dosage compensation: MSL1, a protein that scaffolds MSLc [38, 39], and nucleosome destabilizing factor (Ndf, CG4747), a putative H3K36me3-binding protein that is important for MSLc localization [29]. When we mapped ChIP-seq datasets from these factors, we found that neither gave meaningful signal over the histone gene array (MSL1: Fig. 3B, Ndf/CG4747: Supplemental Fig. 2). This result is not surprising as we previously determined that MSL2 does not target the histone locus in *Drosophila melanogaster* by polytene chromosome immunofluorescence [37].

### Chromatin remodeling candidates

One of the lesser-studied characteristics of the histone locus is the regional chromatin environment. The endogenous histone locus is located on chromosome 2 L, proximal to pericentric heterochromatin. Despite this proximity, histone expression rapidly increases at the start of G1 in preparation for DNA synthesis during S phase, and quickly ceases upon G2 [4], indicating that chromatin remodeling is likely critical in precisely controlling histone gene expression. We therefore hypothesized that chromatin remodeling factors target the histone locus. We chose the following candidates because of their association with chromatin or role in chromatin remodeling: centrosomal 190 kDa protein (CP190), an insulator protein that impacts enhancer-protein interactions and stops the spread of heterochromatin [40]; Gcn5, a lysine acetyltransferase critical for oogenesis and morphogenesis [41]; CCCTC-binding factor (CTCF), a genome architectural protein [42]; Posterior sex combs (Psc), a polycomb-group gene [43]; and Suppressor 12 of zeste 12 (su(z)12), a subunit of polycomb repressive complex 2 [44].

After identifying relevant ChIP-seq datasets (Table 1), we used our analysis pipeline to map data to the histone gene array. We observed that none of the above chromatin remodeling candidates gave meaningful signal over the histone gene array (CP190: Fig. 3C, all others: Supplemental Fig. 2). We were especially surprised that CP190 did not target the histone array. CP190 binds promoter regions, aids enhancer-promoter interactions, and halts the spreading of heterochromatin. Because the histone locus is proximal to pericentric heterochromatin, we hypothesized the presence of CP190 could explain how centromeric heterochromatin does not expand into the histone locus. In addition, CP190 is a member of the Late Boundary Complex (LBC) [45], which also contains the CLAMP protein [46]. We discovered that the LBC binds to the *H3/H4* promoter region in vitro [37]. We were therefore surprised that CP190 does not appear to target the histone gene array, based on the ChIP-seq datasets we analyzed. These data underscore the requirement for visualizing both ChIP and input datasets, rather than just the final normalized trace: although CP190 ChIP-seq does not show enrichment over the histone gene array, bias in the input dataset leads to misleading peaks in the normalized data (Fig. 3C, Supplemental Fig. 2).

### Developmental transcription factor candidates

Zygotic histone biogenesis is critical for the constantly dividing embryo; increased histone expression can lengthen the cell cycle whereas decreased histone levels can shorten the cell cycle [32, 47]. Histone biogenesis is tightly coupled to DNA replication, and excess histones are buffered so as not to interfere with zygotic chromatin [48–50]. We therefore hypothesized that early embryonic transcription factors target the histone locus. We chose the following DNA-binding factors based on their roles in the early embryo: Odd paired (Opa), a pair ruled gene that contributes to morphogenesis [51]; Motif 1 binding protein (M1BP), a transcriptional pausing factor that interacts with the Hox proteins [30, 52]; Hepatocyte nuclear factor 4 (Hnf4), a general developmental transcription factor [53]; Pangolin (Pan), a component of the Wingless signaling pathway [54]; and Pointed (Pnt), a factor that regulates cell proliferation and differentiation during development [55, 56]. When we mapped appropriate ChIP-seq datasets from these factors, none gave meaningful signal over the histone array (Opa: Fig. 3D, M1BP: Fig. 2A, all others: Supplemental Fig. 2).

### Candidates that passed the bioinformatics screen

**Fig. 4:**
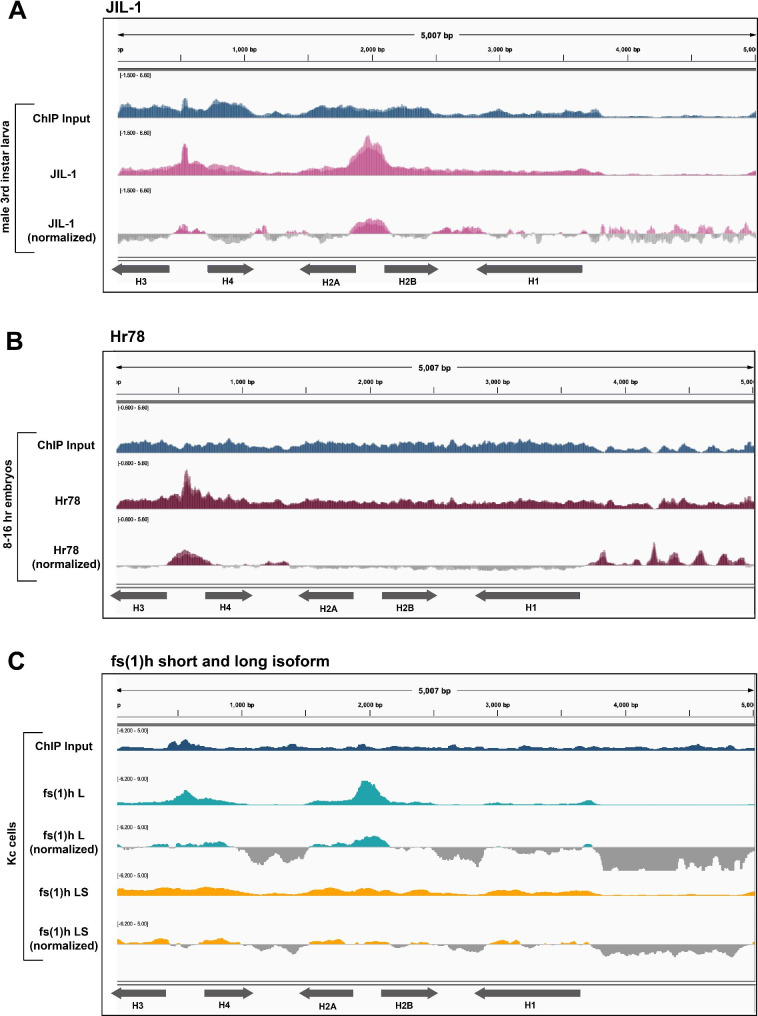
JIL-1, Hr78, and Fs(1)hL localize to the histone gene array. We mapped ChIP datasets for **(A)** JIL-1 (pink, two replicates overlayed, [57]) from male third instar larvae, **(B)** Hr78 (maroon, two replicates overlayed, [73]) from 8–16 h mixed population embryos, and **(C)** the long (L, teal) and short (S, yellow) isoforms of fs(1)h from Kc cells [60] to the histone gene array. We normalized each ChIP-seq dataset to its respective input (blue)

We found several factors that exhibited distinct, meaningful localization patterns to the histone gene array and therefore warrant further investigation (Fig. 4). First, we used our bioinformatics pipeline to map a ChIP-seq dataset for the kinase JIL-1, which is responsible for phosphorylating serine 10 on histone 3 [57, 58]. We observed JIL-1 localizing to the histone gene array, specifically to the *H2A/H2B* promoter (Fig. 4A). We observed an additional sharp peak at the *H3/H4* promoter, but this peak is likely an artifact of short read lengths from the dataset and overlaps with a perfect, long GA-repeat sequence in the *H3/H4* promoter (Fig. 1A, Supplemental Fig. 1). JIL-1 is a DNA-binding factor that associates with the Maleless helicase and MSL1, two members of MSLc [58]. In addition to CLAMP performing a role in histone biogenesis, it also plays a role in dosage compensation and associates with MSLc [59].

We also observed hormone-like receptor 78 (Hr78) localize to the *H3/H4* promoter (Fig. 4B). Finally, we mapped two isoforms of female sterile (1) homeotic (fs(1)h; the *Drosophila* homolog of BRD4). The long and short isoforms of fs(1)h have distinct binding profiles, but both are assumed to have a role in chromatin architecture [60]. We observed that the long isoform, but not the short isoform, localizes to both the *H2A/H2B* and the *H3/H4* promoters (Fig. 4C). Interestingly, Kellner et al. (2013) inferred that the fs(1)h long isoform has a unique role in chromatin remodeling by interacting with specific insulator proteins, including CP190, which did not pass our screen (Fig. 3C).

### Hox factors localize to the ***Drosophila*** histone gene array when overexpressed in cell culture

**Fig. 5:**
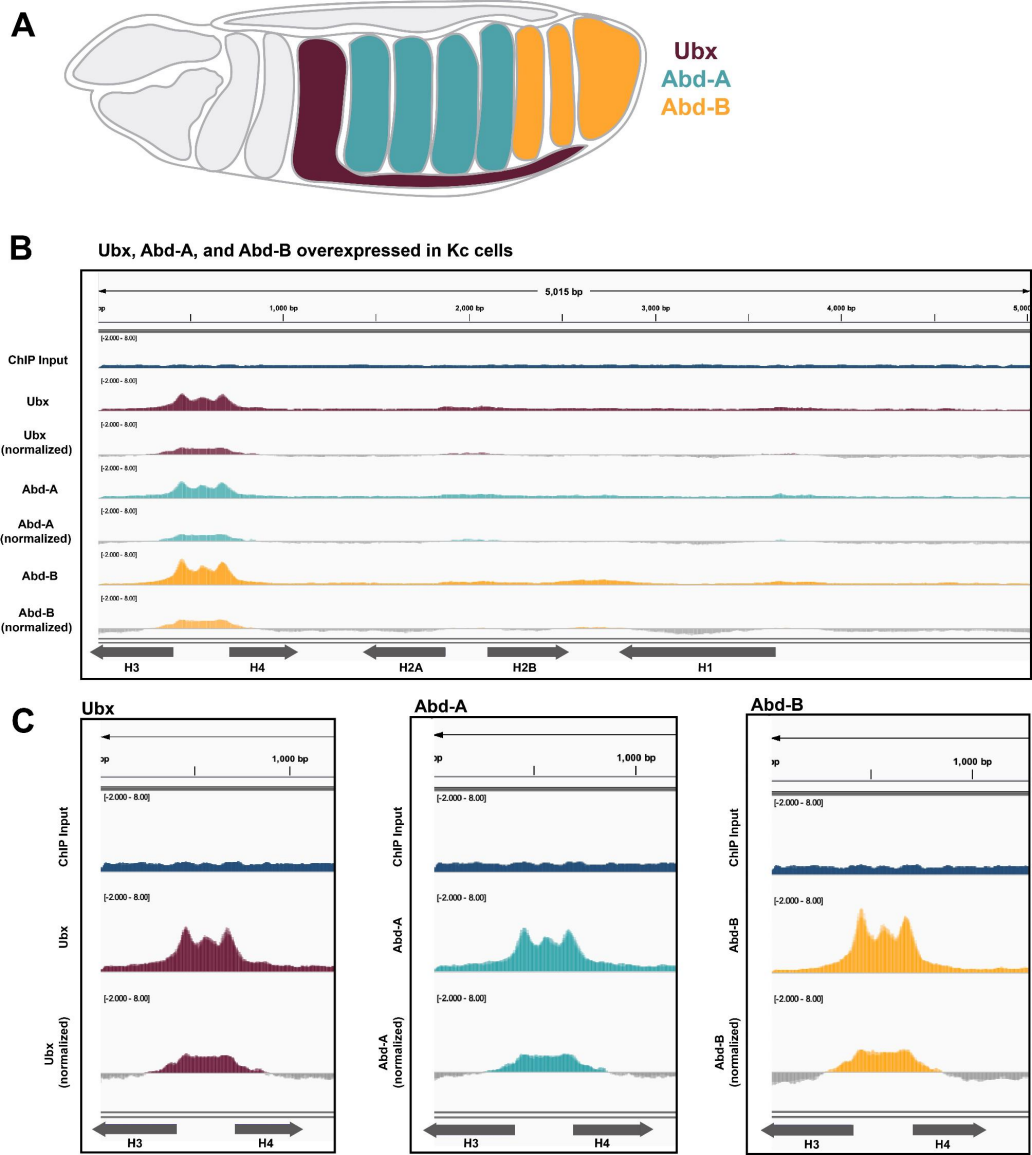
Hox factors Ubx, Abd-A, and Abd-B localize to the histone array. **(A)** Diagram of relative tissue expression patterns for Ubx (maroon), Abd-A (teal) and Abd-B (yellow). **(B)** We aligned ChIP-seq datasets from Kc cells expressing Ubx (maroon, two replicates overlayed, [63] ), Abd-A (teal, two replicates overlayed, [63] ), and Abd-B (yellow, two replicates overlayed, [63] ) to the histone gene array. We normalized each ChIP-seq dataset to the provided input (blue, two replicates overlayed, [63] ). **(C)** Enlarged signal from (B) of Ubx (maroon), Abd-A (teal), and Abd-B (yellow) over the *H3/H4* promoter

Hox factors (Fig. 5A) are critical for developmental processes like morphogenesis, in which cells are constantly dividing and therefore require a near constant supply of histones [4]. Histone biogenesis is critical within the first few hours of *Drosophila* development [32, 47]. We therefore investigated histone array localization patterns of transcription factors that act during early development, including Hox factors. We identified a publicly available dataset (Table 1) in which Beh et al. (2016) individually expressed the three Bithorax complex Hox proteins, Ultrabithorax (Ubx), Abdominal-A (Abd-A), and Abdominal-B (Abd-B), in Kc167 cells and performed ChIP-seq. We used our analysis pipeline to map the Ubx, Abd-A, and Abd-B ChIP-seq datasets to the histone gene array and observed striking localization to the *H3/H4* promoter (Fig. 5B-C). We conclude that when overexpressed in cultured cells, Ubx, Abd-A, and Abd-B all target the histone gene array by ChIP-seq. 

**Fig. 6:**
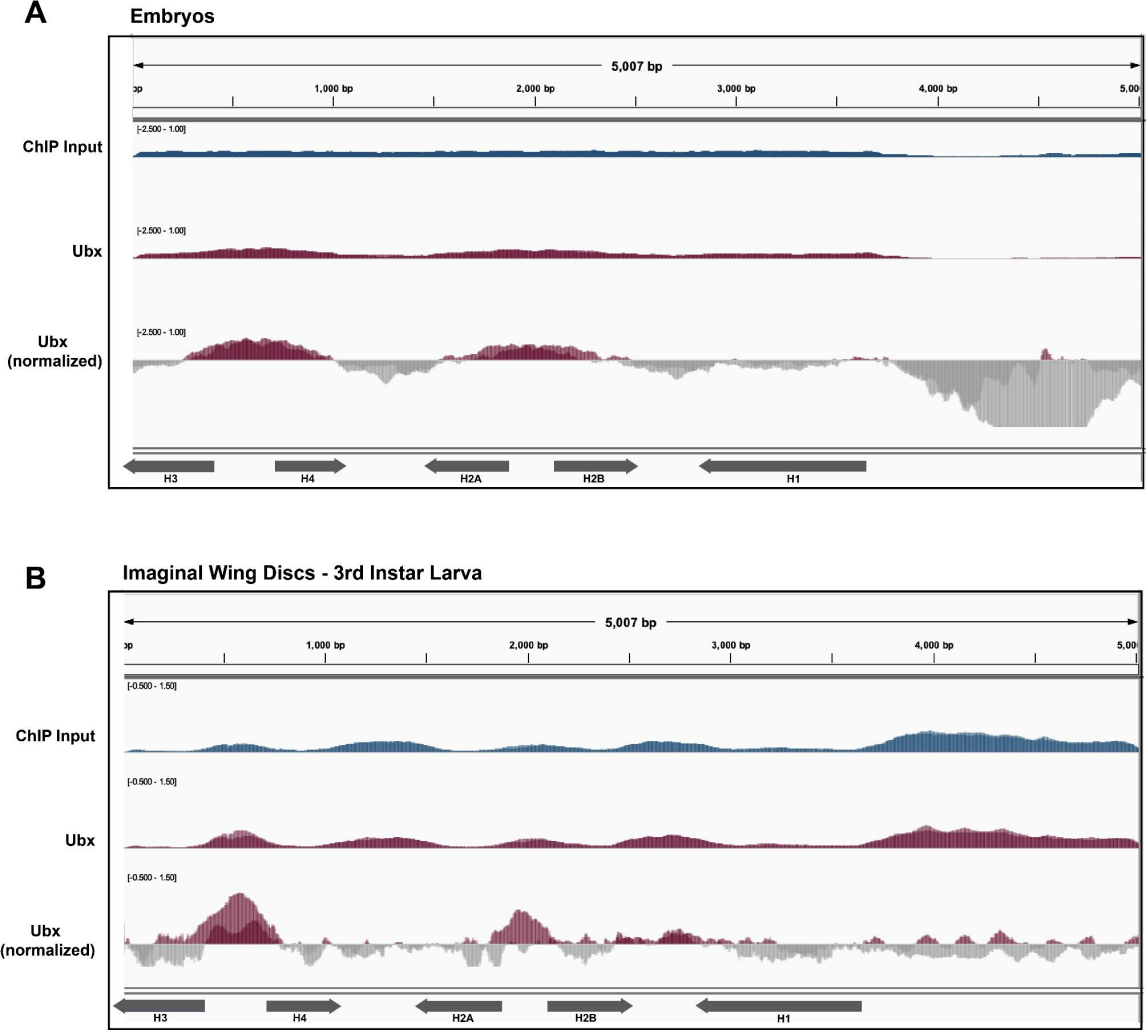
Ubx localizes to the *H3/H4* promoter in embryos and 3rd instar larva. We mapped Ubx ChIP-seq datasets from **(A)** mixed population embryos (maroon, two replicates overlayed, [77] ) and **(B)** imaginal wing discs in third instar larva (maroon, two replicates overlayed, [78] ) to the histone gene array. We normalized ChIP-seq datasets to the provided inputs (blue, two replicates overlayed)

Because our Hox factor observation (Fig. 5) could be an artifact of overexpression in cultured cells, we identified two additional Ubx ChIP-seq datasets from 0 to 16 h embryos and third instar larval imaginal discs (Table 1). We used our pipeline to map these data to the histone gene array and observed that Ubx targets the *H3/H4* promoter and, to a lesser extent, the *H2A/H2B* promoter (Fig. 6). We conclude that Ubx targets the histone gene array at various developmental stages and in various tissues and is therefore a promising candidate for future wet-lab research designed to validate these bioinformatic observations.

To further investigate the relationship between Hox factors and the histone locus, we identified three additional datasets for Hox proteins and Hox cofactors. There are two Hox gene complexes in *Drosophila*: the Bithorax complex (which includes Ubx, Abd-A, and Abd-B) and the Antennapedia complex. We first mapped ChIP-seq data for Antennapedia (Antp) [61] but did not observe robust localization to the histone gene array (Supplemental Fig. 2). We next mapped ChIP-seq datasets for the Hox cofactors extradenticle (Exd) and Homothorax (Hth) [61]. Exd and Hth associate with the hexapeptide motif in Hox proteins and form heterodimers to impact Hox binding specificity to their gene targets [62, 63]. We observed that neither Exd nor Hth gave meaningful ChIP signal over the histone gene array (Supplemental Fig. 2).

### Power and limitations of the screen

The range of results from our candidate screen demonstrates both the power and limitations of our bioinformatics pipeline. In total, we analyzed datasets for 27 different DNA-binding factors and produced 9 candidates that warrant further wet lab investigation. Despite the power of this screen, we are limited by the availability of public datasets. Characteristics of these datasets, such as quality of reads, read length, and inclusions of controls such as inputs are based on the original experimental design and research. Furthermore, we are also restricted by the tissues or genotypes investigated in the original study, limiting the scope of our investigation.

**Fig. 7:**
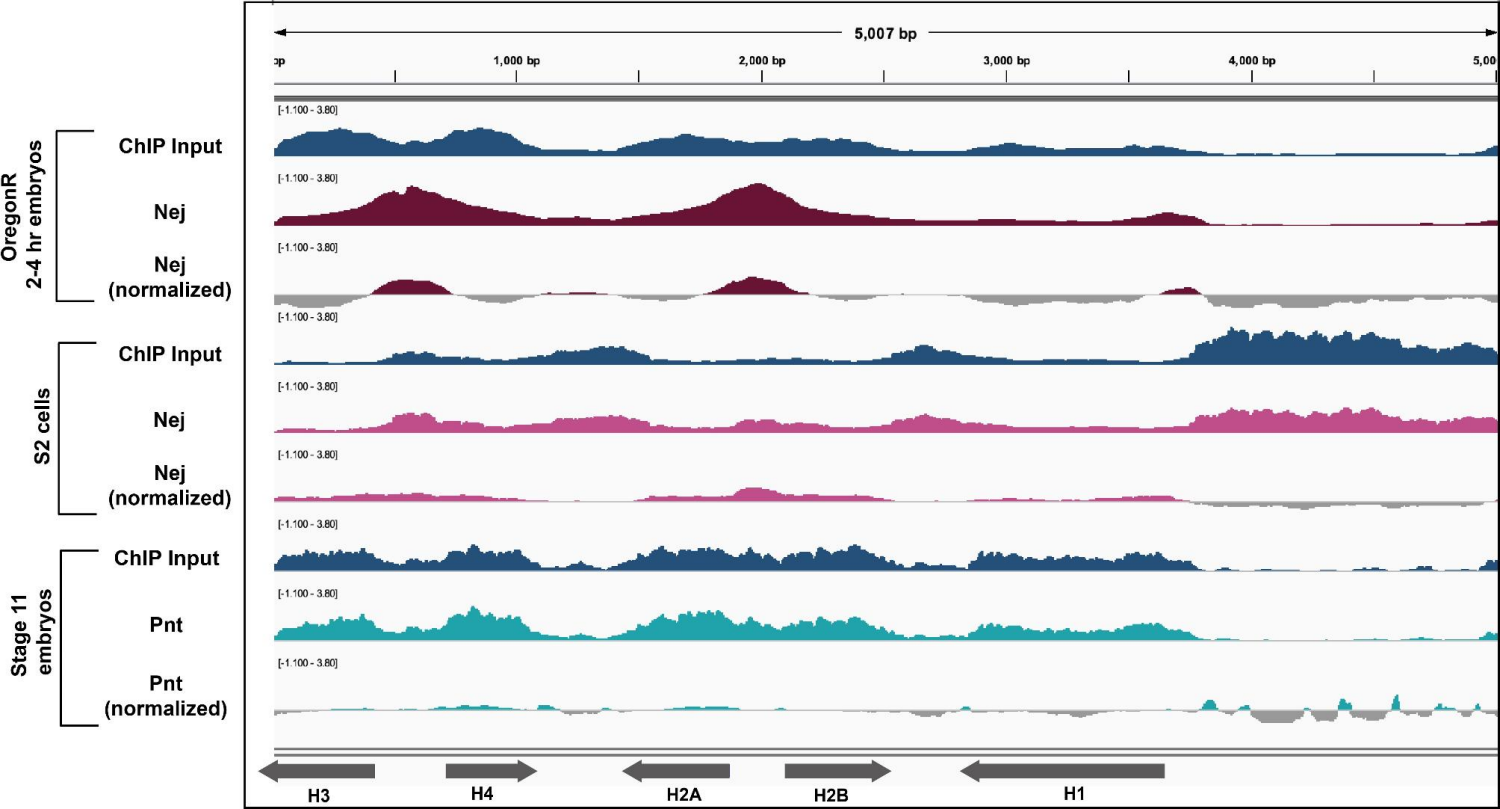
ChIP-seq datasets from different tissues can show different alignment results. We mapped two different ChIP-seq datasets for Nejire (Nej) to the histone gene array. ChIP data from 2–4 h embryos (maroon, one replicate, [74]), showed localization to the *H3/H4* promoter and the *H2A/H2B* promoter, while ChIP-seq data from S2 cells (pink, one replicate, [75] ) showed no localization to the histone gene array. We also aligned ChIP-seq data for Pnt from stage 11 embryos [55] to the histone gene array. We normalized the ChIP-seq signals to their respective input signals (blue)

For example, we analyzed several datasets for Nejire (Nej; homolog of mammalian CREB-binding protein (CBP)) and Pointed (Pnt). A previous screen in S2 cells identified Nej and Pnt as potential HLB factors [11]. We investigated two Nej ChIP-seq datasets (Table 1) in which we obtained disparate results. The Nej ChIP-seq dataset from S2 cells did not yield meaningful signal over the histone gene array (Fig. 7, center). In contrast, we investigated a Nej ChIP-seq dataset from early *Drosophila* embryos and observed robust localization to the *H3/H4* promoter, *H2A/H2B* promoter and, to a lesser extent, the *H1* promoter (Fig. 7, top). From these observations, we conclude that Nej likely targets the histone gene array in embryos and would therefore be a strong candidate for future wet-lab studies to validate this observation. Additionally, we mapped a Pnt ChIP-seq dataset from Stage 11 embryos (Table 1) and observed that Pnt does not give meaningful signal over the histone gene array (Fig. 7, bottom).

Our Pnt and Nej observations demonstrate how our screening approach is powerful but limited by data availability and experimental variables.

## Discussion

To broaden our understanding of factors that impact histone biogenesis in *Drosophila melanogaster*, we conducted a candidate-based bioinformatics screen for DNA-binding factors that localize to the histone gene array. Although many HLB factors are known, it is likely that there are many other factors critical for histone biogenesis that have yet to be identified, since several have been discovered by chance in the past few years including CLAMP [9], Winged-Eye (WGE; [64]), and Myc [21]. To begin to close this gap in knowledge, we chose 27 factors based on their roles in chromatin remodeling, dosage compensation, development, and interaction with known HLB factors, hypothesizing that these represent strong candidates for novel HLB factors. As our screen is limited by availability of relevant datasets, it will likely produce both false positives and negatives. Additionally, because we used a targeted screening approach by investigating factors with relevant functions and at relevant developmental timepoints to histone gene expression, we expected more positive hits than we would find using completely unbiased screen. Given our starting pool of 27 factors, we were pleased to produce 9 candidates for potential HLB factors. We envision that the final 9 candidates that passed our qualitative bioinformatics analysis will be investigated through future wet lab experiments [9, 19, 65].

We validated our bioinformatics pipeline by investigating TRF2, a general transcription factor known to target the histone genes [8], and confirmed that TRF2 binds to the TATA-less *H1* promoter. Isogai et al. (2007) determined that TBP, another general transcription factor, targets the TATA-containing *H3/H4* and *H2A/H2B* promoters. We expanded this observation by investigating TBP-associated factors TAF1, TFIID, and TFIIF. We discovered that all of these general transcription factors target the histone gene array, further validating our pipeline.

We also discovered that the localization of some factors, such as Nej, to the histone gene array is tissue specific. Nej emerged from a proteomic screen for factors involved in HLB activation in cultured cells [11]. However, Nej ChIP-seq from cultured cells did not give meaningful signal over the histone gene array, whereas embryo ChIP-seq showed Nej at histone promoters. These observations denote limitations of our screening technique: we are hindered by the availability and quality of datasets for candidate proteins in specific tissues, genotypes, and conditions.

We initially identified several categories of candidate factors, some of which produced positive hits whereas some did not. For example, Scm, which may interact with the confirmed HLB scaffolding factor Mxc [34, 35, 66], did not show meaningful signal over the histone gene array and therefore we determined that it likely does not target the histone genes.

We also investigated factors involved in dosage compensation, including MSL1, Ndf/CG4747, and JIL-1, because the HLB factor CLAMP plays a key role in male X-chromosome activation. MSL2 was identified in an unbiased proteomics-based HLB candidate screen in cultured cells [11], and we recently discovered that MSLc targets one of the two histone loci in *Drosophila virilis* in salivary gland polytene chromosomes [37]. Although neither MSL1 nor Ndf localized to the histone gene array, JIL-1 robustly localized to the histone gene array.

Of note, the ChIP-seq datasets for MSL1 were produced from S2 cells, the Ndf datasets were from both male and female larvae, and the JIL-1 dataset came specifically from male third instar larvae. MSL1 and Ndf may target the histone gene array in other tissues or only in embryos, representing potential false negatives in our bioinformatics screen. However, JIL-1 is a more generalized kinase that is responsible for phosphorylating serine 10 on histone 3 across the genome, not just on the male X-chromosome [57, 58, 67]. JIL-1 may therefore be present at the histone locus independent of its role in dosage compensation by contributing to the epigenetic landscape of the locus. Taken together, our results indicate that dosage compensation and histone gene expression are likely distinct regulatory events, and the majority of factors are not shared between these processes in *Drosophila melanogaster*.

One of the lesser studied characteristics of the histone locus is the local chromatin environment and how epigenetic marks influence histone gene expression. We chose CP190, Gcn5, Psc, Pangolin, and su(z)12 as chromatin remodeling candidates that might target the histone genes, but after mapping relevant datasets, none of these candidate chromatin remodelers targets the histone gene array. We did, however, discover that the long isoform of fs(1)h (fs(1)hL) robustly localizes to the histone gene array. Fs(1)hL has a unique role in chromatin remodeling that differs from the short isoform, as it associates with insulator proteins, including CP190 [60]. Since the histone locus is situated near heterochromatin, it is possible that insulators prevent spreading of heterochromatin into the histone locus. CP190 was also a strong candidate for histone locus association. CLAMP and CP190 share binding profiles at many promoters and each is important for the other’s localization [40]. However, when we mapped a CP190 ChIP-seq dataset from female embryos, we did not observe histone array localization. Based on these observations, we conclude that fs(1)hL is a strong candidate for future wet lab studies. Fs(1)hL and CLAMP may interact with CP190 at the histone locus, in specific tissues, or at precise developmental timepoints that were not captured in the datasets we investigated.

Finally, we explored several developmental transcription factors, because histone biogenesis is critical in the first few hours of *Drosophila* development during rapid zygotic cell divisions. We chose Opa, M1BP, and Hnf4 as candidates. Despite their roles in early development and patterning, these factors did not target the histone gene array. However, we identified Nej as a candidate that targets the histone gene array, specifically in *Drosophila* embryos but not in S2 cells. Nej was previously identified as an HLB candidate through a cell-based proteomics screen [11]. Nej is a histone acetyltransferase, but it has roles in cell proliferation and developmental patterning. Nej could influence the chromatin environment of the histone locus during key times in development or in tissues that are constantly dividing where histone proteins would be needed. Because of the roles Nej plays in general developmental processes, it is a strong candidate for future wet lab studies.

We were surprised to discover that the Hox proteins Ubx, Abd-A and Abd-B, all localize to the histone array when overexpressed in Kc cells. Specifically, these factors all target the *H3/H4* promoter. This ~ 300 bp promoter is unique within the 5 Kb histone gene array; it is the minimal sequence required for Mxc localization and HLB formation [19] and contains critical GA-repeat *cis*-elements targeted by CLAMP [9]. The CLAMP-GA-repeat interaction promotes recruitment of histone-locus specific transcription factors [9, 20]. To confirm that our observations were not a byproduct of overexpression, we also investigated independent Ubx ChIP-seq datasets prepared from early embryos (0–16 h) and from third instar larval imaginal wing discs. These data confirm that Ubx targets the histone gene array, although the distribution across the array varies between tissues. Ubx, as well as Abd-A and Abd-B, is highly active in the early embryo when histone proteins are needed to organize newly synthesized DNA. Therefore Ubx, Abd-A, and Abd-B could provide a spatial and temporal link between histone biogenesis, cell division, and embryo morphogenesis.

With 9 out of 27 hits from our screen emerging as strong candidates for future studies, our screen has proven to be a powerful tool to identify candidates for DNA-binding factors that target the histone gene array. Controls are specifically important to our pipeline because relative peaks at a given location do not always represent true localization. Our negative hits show a range of different negative signals displayed in Fig. 3. In some cases, we saw clear enrichment for open chromatin regions over promoters and/or gene bodies, but did not characterize these factors as hits based on our qualitative analysis criteria. These regions can be overrepresented in the ChIP sequencing experiment as a whole and, therefore, do not reflect where the DNA-binding factor is truly localizing. This phenomenon is best demonstrated when looking at inputs that also show enrichment over open chromatin or gene bodies as shown in Supplemental Fig. 2. Inputs between datasets can be highly variable and, because they are used in the normalization process, can bias the final visualization.

The HLB was discovered by Liu and Gall only seventeen years ago [68]. Since then, novel HLB factors have largely been discovered one at a time by chance. Proteomic screens identified several new candidates but also failed to identify known factors, including CLAMP [11], indicating the screens are far from saturated. A comprehensive inventory of HLB factors is necessary to establish a thorough mechanism of histone biogenesis. Histone regulation is especially critical in the early animal embryo: excess histones drive extra, asynchronous mitotic cycles, whereas depletion of maternal histones lengthens cell divisions in *Drosophila* embryos [32]. The timing of important early developmental events such as the mid-blastula transition is influenced by histone to DNA ratios [47]. Histone levels also affect pre-mRNA splicing in human cells [69], and *H1* isoform loss-of-function mutations are associated with B cell lymphomas [70]. Factors that influence histone biogenesis likely contribute to these developmental and disease phenotypes.

## Conclusions

Here we present a candidate-based screen for novel histone locus-associating factors. Our screen was largely driven by the undergraduate student coauthors in two stages: first, we identified strong candidates based on their established or inferred roles; second, we identified and mapped relevant ChIP-seq datasets to the histone gene array. A similar recent bioinformatic screen searched through thousands of datasets and hundreds of hematopoietic transcription factors for those associated with the repetitive mammalian rDNA array. This analysis identified numerous candidate transcription factors but required intensive computational pairwise comparisons and thresholding [71]. Another recent screen searched through 1200 chromatin proteins and post-translational modifications to identify those associated with repetitive human centromeres [72]. We instead chose an informed, narrow list of initial candidates and identified 9 out of 27 that we will prioritize for future wet lab studies. Our results not only identify factors that may be involved in histone biogenesis, but also demonstrate the power of a candidate-based bioinformatics screen driven by students.

### Electronic supplementary material

Below is the link to the electronic supplementary material.

Supplementary Material 1

## Data Availability

The authors affirm that all datasets used in the screen are available on GEO (Gene Expression Omnibus). All GEO accession numbers and runs from the SRA run selector are specified in **Table 1**.
