# Supplementary material for: A bioinformatics screen reveals hox and chromatin remodeling factors at the Drosophila histone locus
Source: BMC Genom Data. 2023 Sep 21;24:54. doi: 10.1186/s12863-023-01147-0 (PMC10515271; doi:10.1186/s12863-023-01147-0)
Supplement: Supplementary file 1 — Supplementary Material 1 [file 12863_2023_1147_MOESM1_ESM.pdf]

Supplemental Figures

Hodkinson, Smith, *et al.* **A bioinformatics screen reveals Hox and chromatin remodeling factors at the *Drosophila* histone locus**

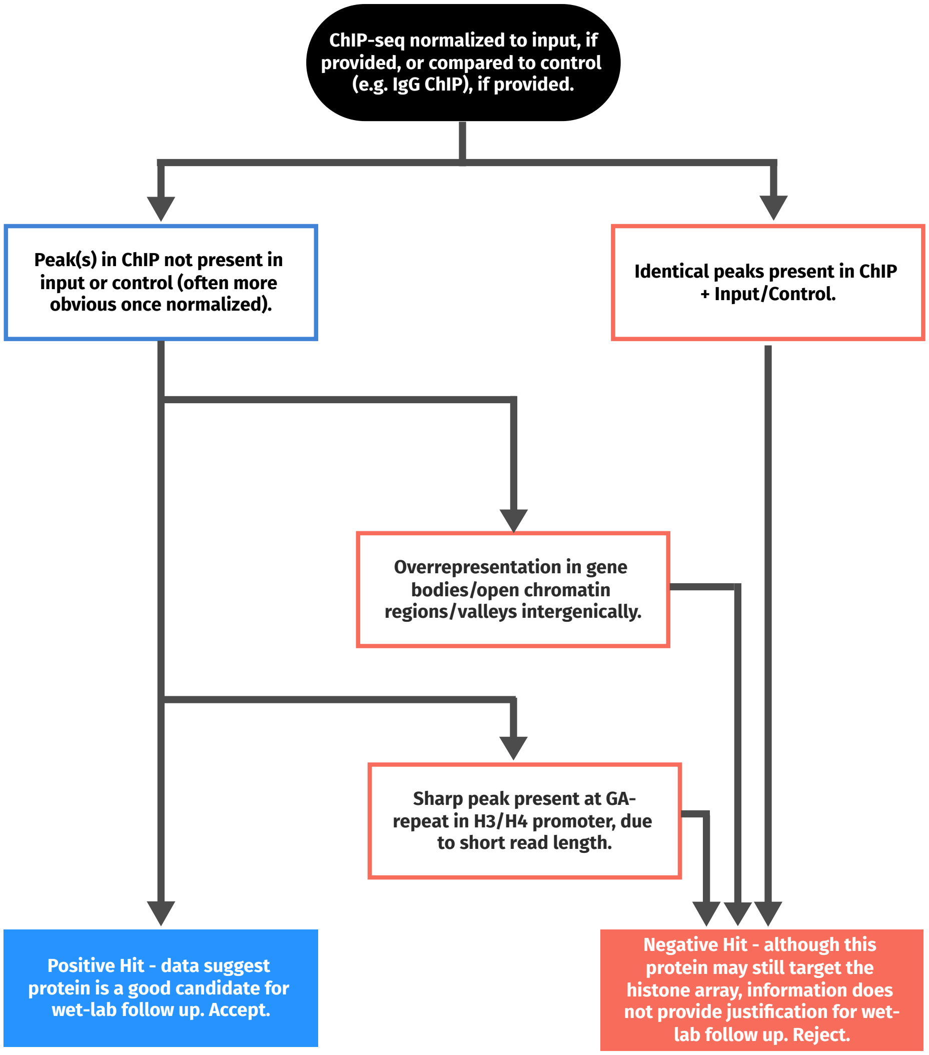

Supplemental Figure 1: Qualitative assessment for scoring candidates as positive or negative.

Supplemental Figure 2: Factors considered negative hits.

Antennapedia

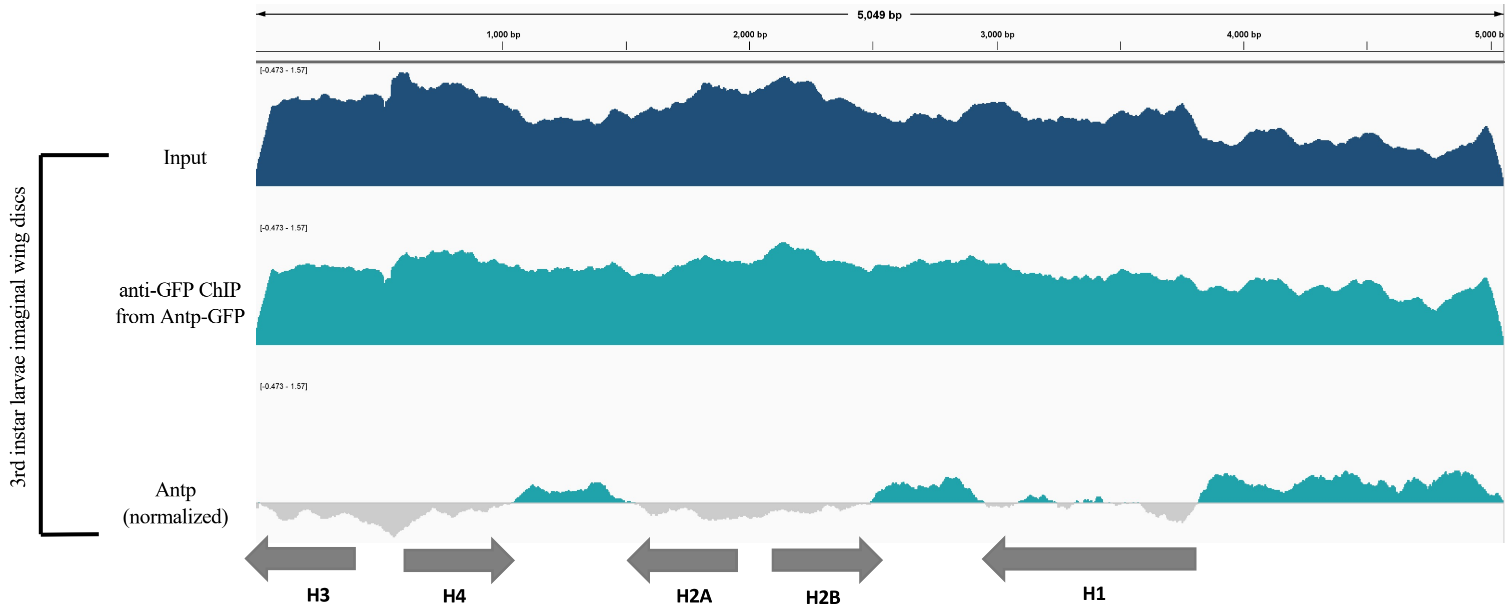

We mapped Antennapedia (Antp) ChIP-seq (cyan) and input (navy) data (Kribelbauer, *et al.* 2020) from 3rd instar larvae imaginal wing discs to the histone gene array. Experiment used an anti-GFP antibody to immunoprecipitate Antp-GFP. Antp does not show convincing localization to the histone gene array when compared to input.

CTCF

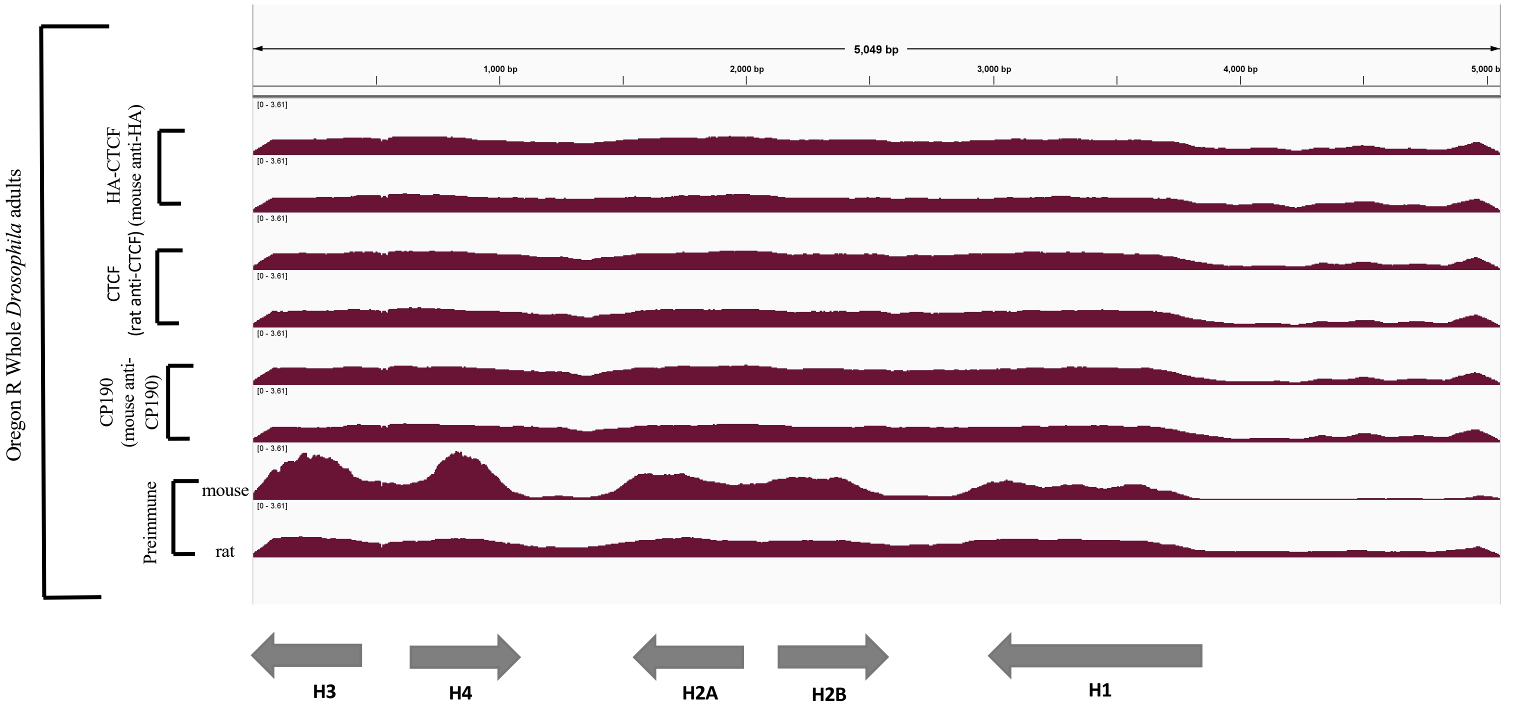

We mapped CTCF-HA ChIP, CP190 ChIP, and preimmune ChIP-seq data (Kyrchanova, *et al.* 2021) from OregonR whole *Drosophila* adults to the histone gene array. CTCF and CP190 do not show convincing localization to the histone gene array when compared to preimmune.

Extradenticle

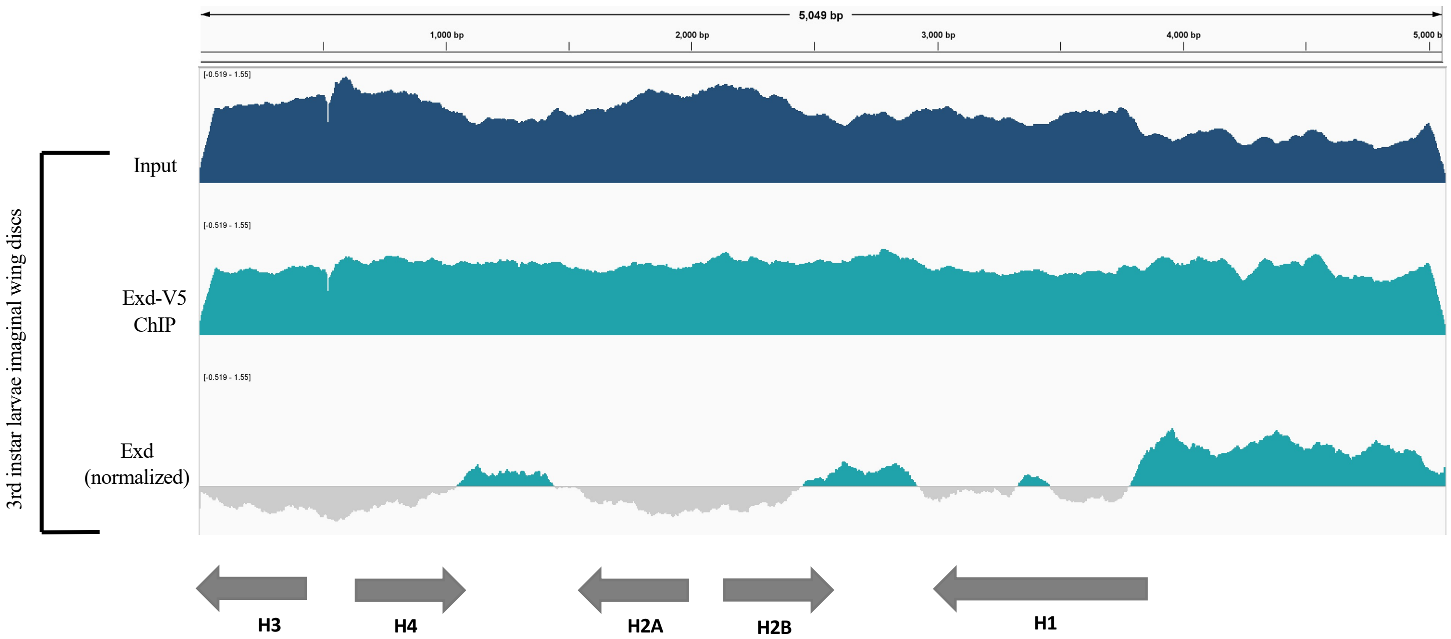

We mapped Extradenticle-V5 (Exd) ChIP-seq (cyan) and input (navy) data (Kribelbauer, *et al.* 2020) from 3rd instar larvae imaginal wing discs to the histone gene array. Experiment used an anti-V5 antibody to immunoprecipitate Exd-GFP. Exd does not show convincing localization to the histone gene array when compared to input.

Gcn5

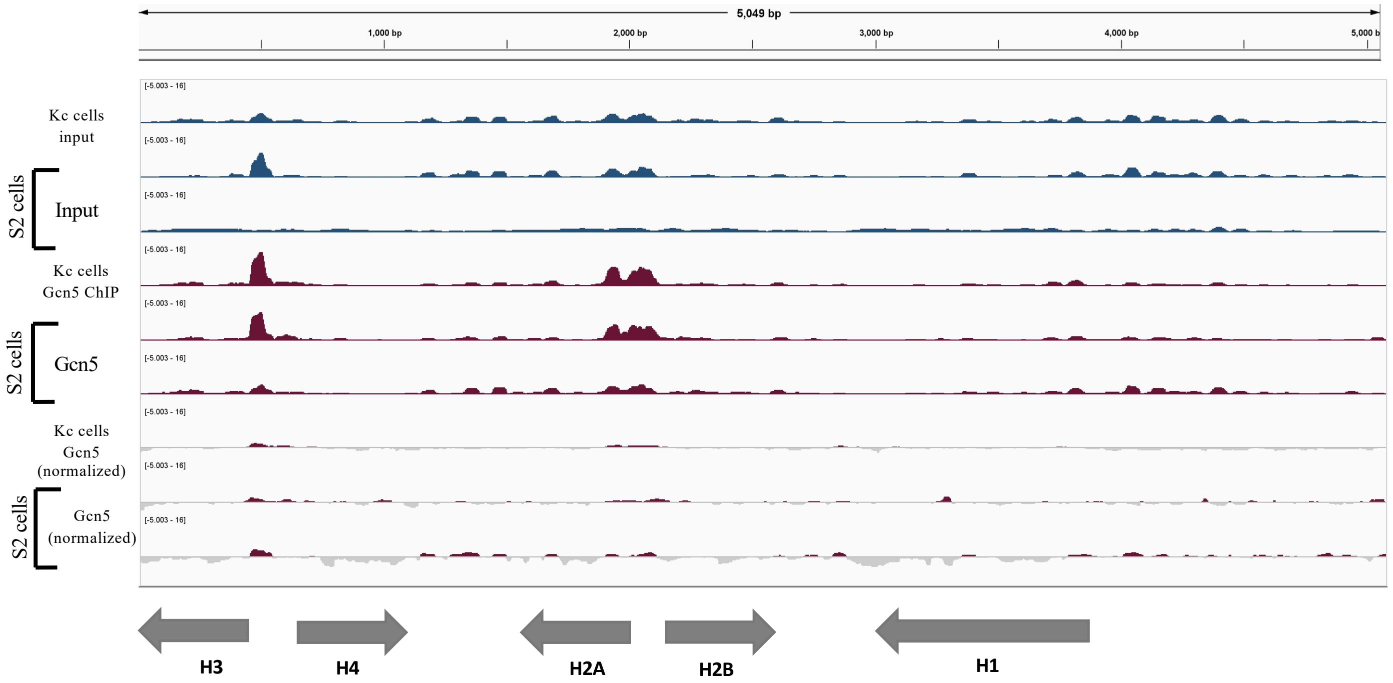

We mapped Gcn5 acetyltransferase (Gcn5) ChIP-seq (maroon) and input (navy) data (Ali, *et al.* 2017) from both Kc cells (one replicate) and S2 cells (two replicates) to the histone gene array. Gcn5 does not show convincing localization to the histone gene array when compared to the corresponding input.

Hepatocyte nuclear factor 4

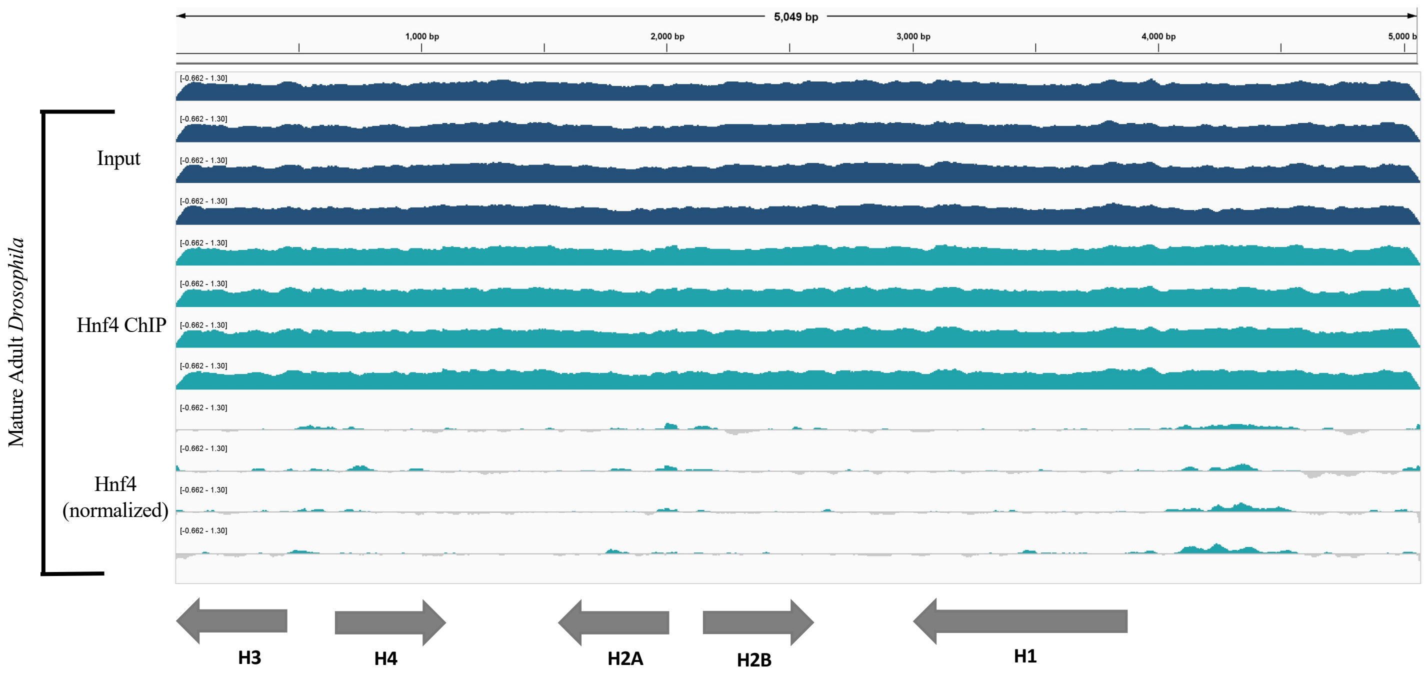

We mapped Hepatocyte nuclear factor 4 (Hnf4) ChIP-seq (cyan) and input (navy) data (Thummel, *et al.* 2015) from whole mature adult *Drosophila* to the histone gene array. Hnf4 does not show convincing localization to the histone gene array when compared to input.

Homothorax

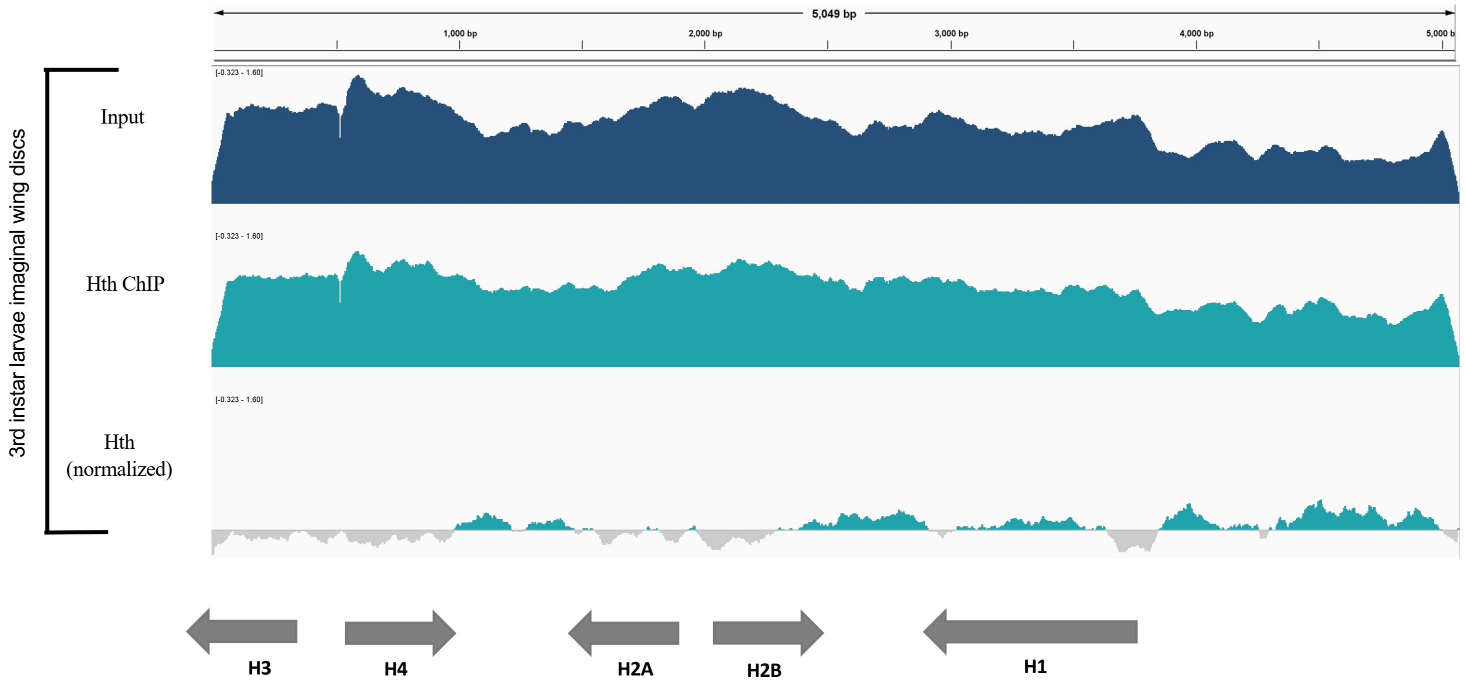

We mapped Homothorax (Hth) ChIP-seq (cyan) and input (navy) data (Kribelbauer, *et al.* 2020) from 3rd instar larvae imaginal wing discs to the histone gene array. Hth does not show convincing localization to the histone gene array when compared to input.

Nucleosome-destabilizing factor/CG4747

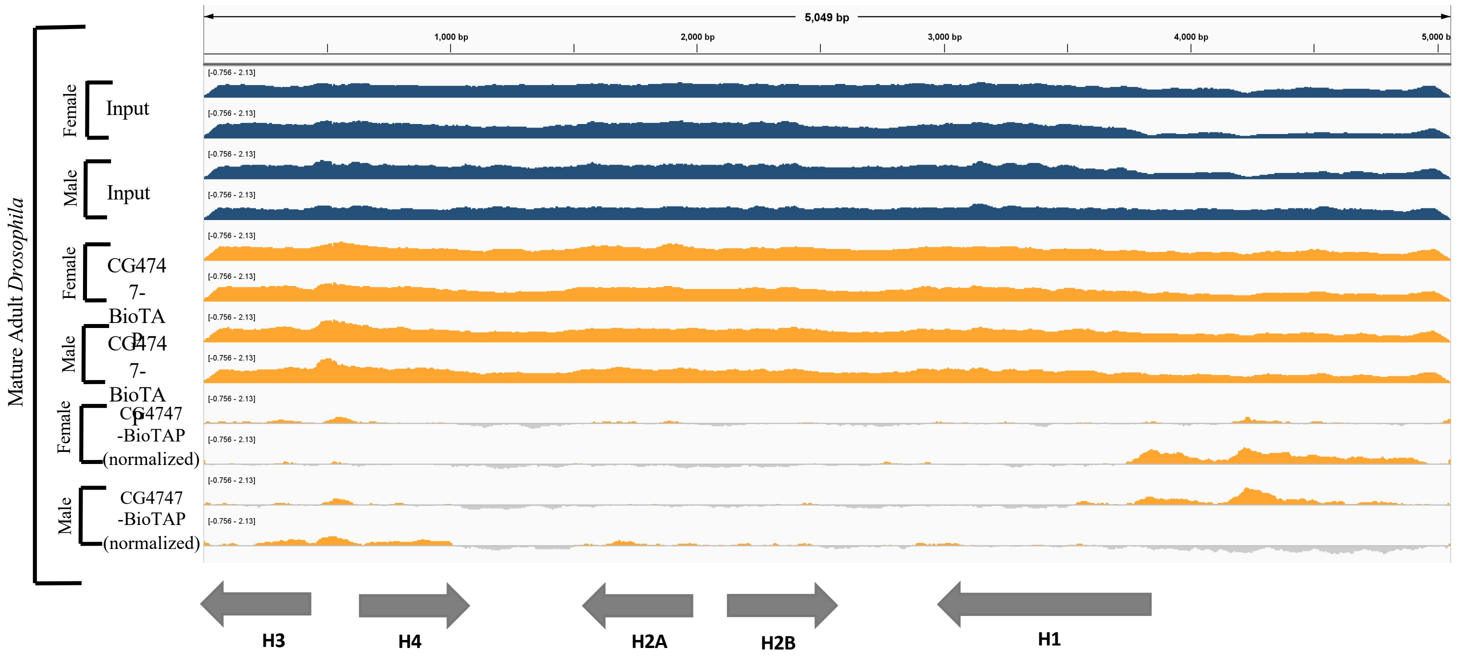

We mapped Nucleosome-destabilizing factor-BioTAP (Ndf/CG4747) ChIP-seq (yellow) and input (navy) data (GSE42025) from whole mature adult *Drosophila* to the histone gene array. Experiment used an anti-Bio-TAP antibody in animals expressing CG47474-BioTAP. Ndf/CG47474 does not show convincing localization to the histone gene array when compared to input.

Pangolin

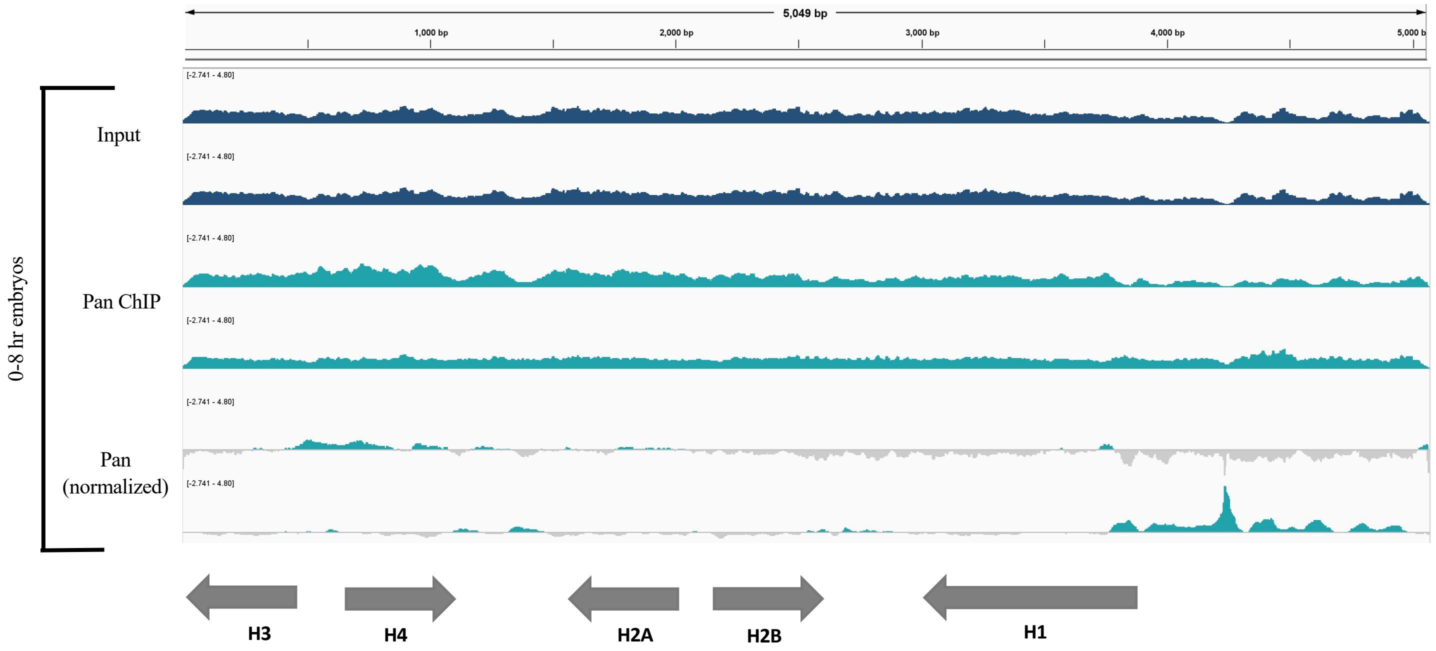

We mapped Pangolin (Pan) ChIP-seq (cyan) and input (navy) data (ModENCODE) from 0-8 hr embryos from the *y,cn,bw,sp* genotype to the histone gene array. Pan does not show convincing localization to the histone gene array when compared to input.

Posterior Sex Combs

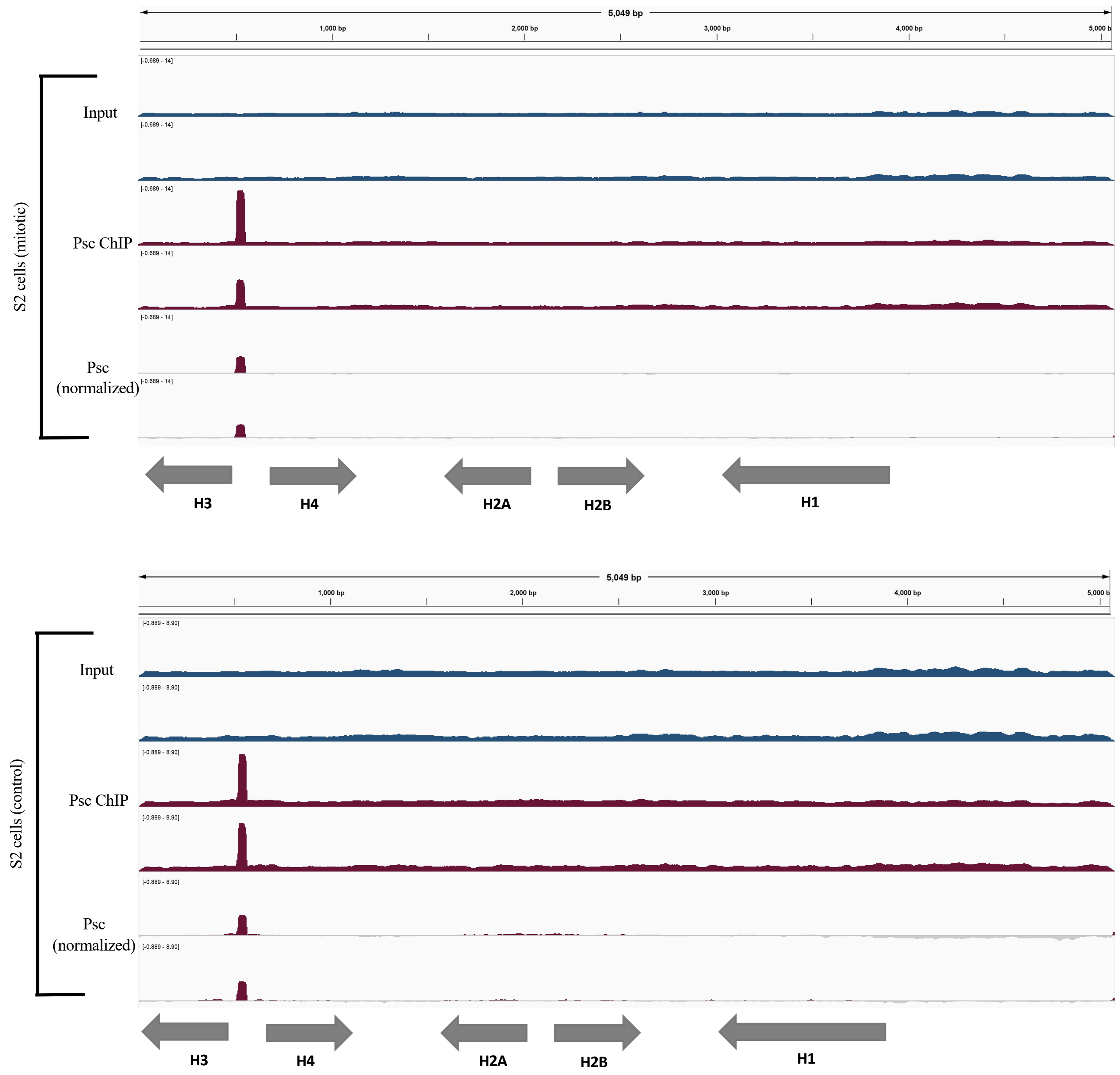

We mapped Posterior sex combs (Psc) ChIP-seq (maroon) and input (navy) data (Follmer *et al*., 2012) from mitotic (top) and control (bottom) S2 cells to the histone gene array. While a sharp peak is apparent in the *H3/H4* promoter, this corresponds to a perfect GA-repeat and is due to the short read length (40 bp) of the datasets. Psc does not show convincing localization to the histone gene array when compared to the corresponding input.

Scm (embryos)

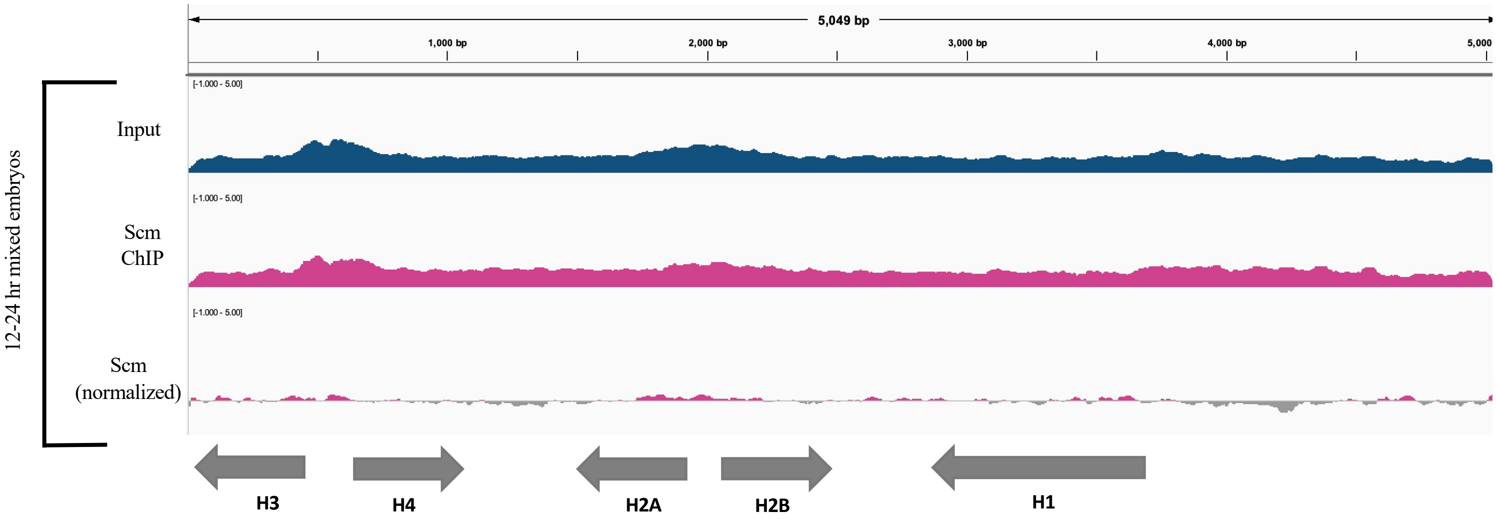

We mapped Scm ChIP-seq (pink) and input (navy) data (Kang H *et al.* 2015) from 12-24 hr mixed population embryos to the histone gene array. Scm does not show convincing localization to the histone gene array when compared to input.

Suppressor of zeste 12

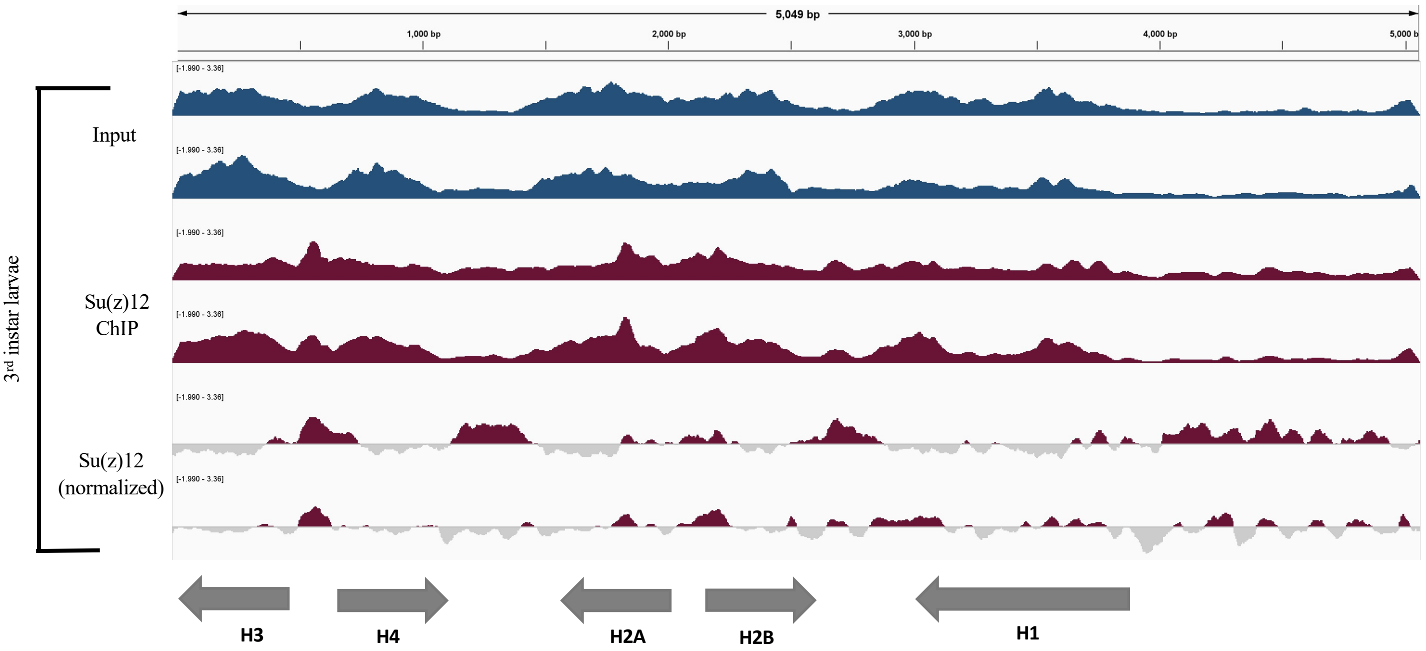

We mapped Suppressor of zeste 12 (Su(z)12) ChIP-seq (maroon) and input (navy) data (Herz *et al.*, 2012) from 3rd instar larvae to the histone gene array. Su(z)12 does not show convincing localization to the histone gene array when compared to input.

Supplemental Table 1

Candidate	Category	Rationale	Tissue/Timing	Localization	Region
**Abd-A**<br>Abdominal A	Early development transcription factor	Continuous expression post 0-2hour developmental stage,<br>with highest RNA expression levels between 2-4hrs (73,79)	Kc cells	Yes	*H3/H4* promoter
**Abd-B**<br>abdominal B	Early development transcription factor	Continuous expression post 0-2hour developmental stage,<br>with highest RNA expression levels between 2-4hrs (73,79)	Kc cells	Yes	*H3/H4* promoter
**Antp**<br>Antennapedia	Early development transcription factor	Expressed in the early embryo (73,79)	Imaginal wing disk		
**CTCF**<br>CCCTC-binding factor	Chromatin structure/remodeler	Key chromatin architecture protein, serves as an insulator and allows for distant DNA interacts (42)	Mixed adults		
**CP190**<br>Centrosomal protein 190kD	Chromatin structure/remodeler	Insulates centromeric heterochromatin (40)	Kc/S2 cells		
**Exd**<br>extradenticle	Early development transcription factor	Expressed in the early embryo (73,79)	5-6 days wing		
**Fs(1)h**<br>female sterile (1) homeotic	Chromatin structure/remodeler	short isoform binds at promoters and enhancers and long isoform binds at chromatin insulators<br>(60)	Kc cells	Yes (long isoform only)	*H3/H4* promoter<br>*H2A/H2B* promoter
**Gcn5**	Chromatin structure/remodeler	Acetyltransferase, critical for oogenesis and morphogenesis (41) and associates with insulators (40)	Kc cells		
**Hnf4**<br>Hepatocyte nuclear factor 4	Early development transcription factor	Important for major events in embryogenesis, works in cell metabolism pathways (53)	Mixed adults		
**Hr78**<br>Hormone-receptor-like in 78	Early development transcription factor	Continuous expression throughout embryogenesis and during metamorphosis (73,79)	8-16h embryos	Yes	*H3/H4* promoter
**Hth**<br>homothorax	Early development transcription factor	Expressed in the early embryo (73,79)	Imaginal wing disc		
**JIL-1**	Dosage compensation/ X-chromosome associated factor	Kinase phosphorylation of H3S10<br>Enriched on X chromosome for dosage compensation<br>Serine 10 mark prevents heterochromatin spreading (57,58,67)	3rd instar larvae	Yes	*H2A/H2B* promoter
**M1BP**<br>Motif 1 Binding Protein	Chromatin structure/remodeler	Interacts with TRF2 and related to insulator activity by associating with CP190 (52)	Kc/S2 cells		
**MSL1**<br>Male specific Lethal 1	Dosage compensation factor	Associates with the known HLB factor CLAMP (46,59)	S2 cells		
**Ndf (CG4747)**<br>Nucleosome-destabilizing factor	Dosage compensation associated factor	H3K36me3-binding protein that is important for MSLc localization (29)	larval		
**Nej**<br>Nejire	Hit from previous screen for HLB factors<br>(11)	Acetyltransferase and early developmental transcription factor	2-4 hr embryos<br>S2 cells	Yes (embryos only)	*H3/H4* promoter<br>*H2A/H2B* promoter
**Opa**<br>Odd Paired	Early development transcription factor	Continuous expression post 0-2hour developmental stage,<br>with highest RNA expression levels between 2-4hr when ZGA/ histone gene expression is high (73,79)	3h embryo<br>4h embryo		
**Pan**<br>Pangolin	Early development transcription factor	Expressed in the early embryo/early in development (73,79)	0-8h embryo		
**Pnt**<br>Pointed	Hit from previous screen for HLB factors<br>(11)	Early development transcription factor	Stage 11 embryo		
**Psc**<br>Posterior sex combs	Chromatin structure/remodeler	Polycomb member (43)	S2 cells		

**Scm**<br>Sex comb on midleg	HLB-associated factor	Genetically interacts with known HLB factor Mxc (34,35)	Embryo 12-24h<br>S2 cells		
**su(z)12**<br>suppressor of zeste 12	Chromatin structure/remodeler	Polycomb repressive complex member (44)<br>Highest RNA expression levels during 0-2h and 4-8h development stages (73,79)	3rd instar larvae		
**TAF1**<br>TBP-associated factor 1	General transcription factor	TATA-box-binding protein known to associate with TBP (30)	S2 cells	Yes	*H3/H4* promoter
**TFIIB**<br>Transcription Factor II B	General transcription factor	TATA-box binding protein complex member, known to associate with TBP (31)	OregonR Embyos	Yes	*H3/H4* promoter<br>*H2A/H2B* promoter
**TFIIF**<br>Transcription Factor II F	General transcription factor	TATA-box binding protein complex member, known to associate with TBP (31)	OregonR Embyos	Yes	*H3/H4* promoter<br>*H2A/H2B* promoter<br>*H1* promoter
**TRF2**<br>TATA box binding protein-related factor 2	General transcription factor	TATA-less promoter binding activity at *H1* promoter<br>(8)	S2 cells	Yes	*H1* promoter
**Ubx**<br>Ultrabithorax	Early development transcription factor	Continuous expression post 0-2hour developmental stage,<br>with highest RNA expression levels between 2-4hrs (73,79)	Kc cells,<br>imaginal wing disc<br>embryos	Yes (all)	*H3/H4* promoter

Supplementary Table 1: All candidates categories, function, and tissue details.

Supplemental References:
79. Gramates LS, Agapite J, Attrill H, Calvi BR, Crosby MA, dos Santos G, et al. FlyBase: a guided tour of highlighted features. Genetics. 2022 Apr 1;220(4):iyac035.
